# Modeling and prediction of clinical symptom trajectories in Alzheimer’s disease using longitudinal data

**DOI:** 10.1371/journal.pcbi.1006376

**Published:** 2018-09-14

**Authors:** Nikhil Bhagwat, Joseph D. Viviano, Aristotle N. Voineskos, M. Mallar Chakravarty, Alzheimer’s Disease Neuroimaging Initiative

**Affiliations:** 1 Institute of Biomaterials and Biomedical Engineering, University of Toronto, Toronto, Ontario, Canada; 2 Computational Brain Anatomy Laboratory, Brain Imaging Center, Douglas Mental Health University Institute, Verdun, Quebec, Canada; 3 Kimel Family Translational Imaging-Genetics Research Lab, Campbell Family Mental Health Research Institute, Centre for Addiction and Mental Health, Toronto, Ontario, Canada; 4 Department of Psychiatry, University of Toronto, Toronto, Ontario, Canada; 5 Department of Biological and Biomedical Engineering, McGill University, Montreal, Quebec, Canada; Oxford University, UNITED KINGDOM

## Abstract

Computational models predicting symptomatic progression at the individual level can be highly beneficial for early intervention and treatment planning for Alzheimer’s disease (AD). Individual prognosis is complicated by many factors including the definition of the prediction objective itself. In this work, we present a computational framework comprising machine-learning techniques for 1) modeling symptom trajectories and 2) prediction of symptom trajectories using multimodal and longitudinal data. We perform primary analyses on three cohorts from Alzheimer’s Disease Neuroimaging Initiative (ADNI), and a replication analysis using subjects from Australian Imaging, Biomarker & Lifestyle Flagship Study of Ageing (AIBL). We model the prototypical symptom trajectory classes using clinical assessment scores from mini-mental state exam (MMSE) and Alzheimer’s Disease Assessment Scale (ADAS-13) at nine timepoints spanned over six years based on a hierarchical clustering approach. Subsequently we predict these trajectory classes for a given subject using magnetic resonance (MR) imaging, genetic, and clinical variables from two timepoints (baseline + follow-up). For prediction, we present a longitudinal Siamese neural-network (LSN) with novel architectural modules for combining multimodal data from two timepoints. The trajectory modeling yields two (stable and decline) and three (stable, slow-decline, fast-decline) trajectory classes for MMSE and ADAS-13 assessments, respectively. For the predictive tasks, LSN offers highly accurate performance with 0.900 accuracy and 0.968 AUC for binary MMSE task and 0.760 accuracy for 3-way ADAS-13 task on ADNI datasets, as well as, 0.724 accuracy and 0.883 AUC for binary MMSE task on replication AIBL dataset.

## Author summary

With an aging global population, the prevalence of Alzheimer’s disease (AD) is rapidly increasing, creating a heavy burden on public healthcare systems. It is, therefore, critical to identify those most likely to decline towards AD in an effort to implement preventative treatments and interventions. However, predictions are complicated by the substantial heterogeneity present in the clinical presentation in the prodromal stages of AD. Longitudinal data comprising cognitive assessments, magnetic resonance images, along with genetic and demographic information can help model and predict the symptom progression patterns at the single subject level. Additionally, recent advances in machine-learning techniques provide the computational framework for extracting combinatorial longitudinal and multimodal feature sets. To this end, we have used multiple AD datasets consisting of 1000 subjects with longitudinal visits spanned up to six years for 1) modeling stable versus declining clinical symptom trajectories and 2) predicting these trajectories using data from both baseline and a follow-up visits within one year. From a computational standpoint, we validated that a machine-learning model is capable of combining longitudinal, multimodal data towards accurate predictions. Our validations demonstrate that the presented model can be used for early detection of individuals at risk for clinical decline, and therefore holds crucial clinical utility for AD, as well as, other neurodegenerative disease interventions.

## Introduction

Clinical decline towards Alzheimer’s disease (AD) and its preclinical stages (significant memory concern [SMC] and mild cognitive impairment [MCI]) increases the burden on healthcare and support systems [1]. Identification of the declining individuals *a priori* would provide a critical window for timely intervention and treatment planning. Individual level clinical forecasting is complicated by many factors that includes the definition of the prediction objective itself. Several previous efforts have focused on diagnostic conversion, within a fixed time-window, as a prediction end point (e.g. the conversion of MCI to frank AD onset) [2–11]. Other studies, which model clinical states as a continuum instead of discrete categories, investigate prediction problems pertaining to symptom severity. These studies define their objective as predicting future clinical scores from assessments such as mini-mental state exam (MMSE) and Alzheimer’s Disease Assessment Scale-cognitive (ADAS-cog) [6,12,13]. All of these tasks have proved to be challenging due to the heterogeneity in clinical presentation comprising highly variable and nonlinear longitudinal symptom progression exhibited throughout the continuum of AD prodromes and the onset [14–19].

In the pursuit of predictive biomarkers identification, several studies have reported varying neuroanatomical patterns associated with functional and cognitive decline in AD and its prodromes [7,16,20][14–19][21–25]. The lack of localized, canonical atrophy signatures could be attributed to cognitive reserve, genetics, or environmental factors [21,26] [17,27–30]. As a result, local anatomical features, such as hippocampal volume, may be insufficient for predicting future clinical decline at a single subject-level [16,19,31][14–19]. Thus, models incorporating an ensemble of imaging features, clinical and genotypic information have been proposed [6,8,9]. However, in such multimodal models, the performance gains offered by the imaging data are unclear. Particularly, insight into prediction improvement from magnetic resonance (MR) images is crucial, as it may aid decision making regarding the necessity of the MR acquisition (a relatively expensive, time-consuming, and possibly stressful requirement) for a given subject in the aims of improving prognosis. Furthermore, there is increasing interest in incorporating data from multiple timepoints (i.e. follow-up patient visits), in an effort to improve long-term prognosis. However, this is a challenging task requiring longitudinally consistent feature selection and mitigation of missing timepoints [6,10].

The overarching goal of this work is to provide a longitudinal analysis framework for predicting symptom progression in AD that addresses the aforementioned challenges pertaining to task definition (model output) as well as ensemble feature representation (model input). The contributions of this work are two-fold. First, we present a novel data-driven approach for modeling long-term symptom trajectories derived solely from clustering of longitudinal clinical assessments. We show that the resultant trajectory classes represent relatively stable and declining trans-diagnostic subgroups of the subject population. Second, we present a novel machine-learning (ML) model called longitudinal Siamese network (LSN) for prediction of these symptom trajectories based on multimodal and longitudinal data. Specifically, we use cortical thickness as our MR measure due to its higher robustness against typical confounds, such as head size, total brain volume, etc., compared to local volumetric measures [32] and its previous use in biomarker development and clinical applications in AD [16,21,33,34]. The choice of excluding other potential biomarkers related to AD-progression, such as PET or CSF data in the analysis was based on their invasive acquisition and lack of availability in practice and in the databases leveraged in this work.

We evaluate the performance of trajectory modeling and prediction tasks on Alzheimer’s disease Neuroimaging Initiative (ADNI) datasets (ADNI1, ADNIGO, ADNI2) [http://adni.loni.usc.edu/]. Moreover, we also validate the predictive performance on a completely independent replication cohort from Australian Imaging, Biomarker & Lifestyle Flagship Study of Ageing (AIBL) [http://adni.loni.usc.edu/study-design/collaborative-studies/aibl/]. We compare LSN with other ML models including logistic regression (LR), support vector machine (SVM), random forest (RF), and classical artificial neural network (ANN). We examine the added value of MR information in combination with clinical and demographic data, as well as, the benefit of the follow-up timepoint information towards the prediction task to assist prioritization of MR data acquisition and periodic patient monitoring.

## Materials and methods

### Datasets

ADNI1, ADNIGO, ADNI2, and AIBL datasets were downloaded from Alzheimer’s disease Neuroimaging Initiative (ADNI) database (http://adni.loni.usc.edu/). The Australian Imaging, Biomarker & Lifestyle (AIBL) Flagship Study of Ageing is a collaborative study that shares many common goals with ADNI (http://adni.loni.usc.edu/study-design/collaborative-studies/aibl/). Only ADNI-compliant subjects with at least three timepoints from AIBL were included in this study. For all ADNI cohorts, subjects with clinical assessments from at least three visits, in timespan longer than one year, were included in the analysis. Subjects were further excluded based on manual quality checks after MR preprocessing pipelines (described below). Age, Apolipoprotein E4 (APOE4) status, clinical scores from mini-mental state exam (MMSE) and Alzheimer’s Disease Assessment Scale (ADAS-13), and T1-weighted MR images were used in the analysis. Subject demographics are shown in Table 1, and the complete list of included subjects is provided in S1 File.

**Table 1:** Demographics of ADNI and AIBL datasets. TM: trajectory modeling cohort, TP: trajectory prediction cohort. R: replication cohort.

Dataset	N	Age (years)	APOE4 (n_alleles)	Sex	Dx (at baseline)
**ADNI1 (TM)**	69	74.5	0: 30, 1: 32, 2: 7	M: 51, F: 18	LMCI: 69
**ADNI1 (TP)**	513	75.3	0: 275, 1: 180, 2: 58	M: 289, F: 224	CN: 177, LMCI: 226, AD: 110
**ADNI2 (TP)**	515	72.4	0: 277, 1: 187, 2: 51	M: 281, F: 234	CN: 150, SMC: 26, EMCI: 142, LMCI: 130, AD: 67
**ADNIGO (TP)**	88	71.1	0: 54, 1: 26, 2: 8	M: 47, F: 41	EMCI: 88
**AIBL (R)**	117	71.7	0: 65, 1: 46, 2: 6	M: 61, F: 56	CN: 99, MCI: 18

The ADNI sample comprising pooled ADNI1, ADNIGO, and ADNI2 subjects was used to perform primary analysis comprising trajectory modeling and prediction tasks, whereas AIBL subjects were used as the independent replication cohort for the prediction task. There are a few important differences in the ADNI and AIBL cohorts: ADNI has clinical measures from both MMSE and ADAS-13 scales with visits separate by 12 months or less spanning 72 months; while AIBL collected MMSE data with visits separated by 18 months or more spanning 54 months.

### Preprocessing

T1-weighted MR images were used in this study. First, the MR images were preprocessed using the bpipe pipeline (https://github.com/CobraLab/minc-bpipe-library/) comprising N4-correction [35], neck-cropping to improve linear registration, and BEaST brain extraction [36]. Then, the preprocessed images were input into the CIVET pipeline [37–40] to estimate cortical surfaces measures at 40,962 vertices per hemisphere. Lastly, cortical vertices were grouped into 78 region of interests (ROIs) based on the Automated Anatomical Labeling (AAL) atlas [41] that were used to estimate mean cortical thickness across each ROI.

### Analysis workflow

**Fig 1:**
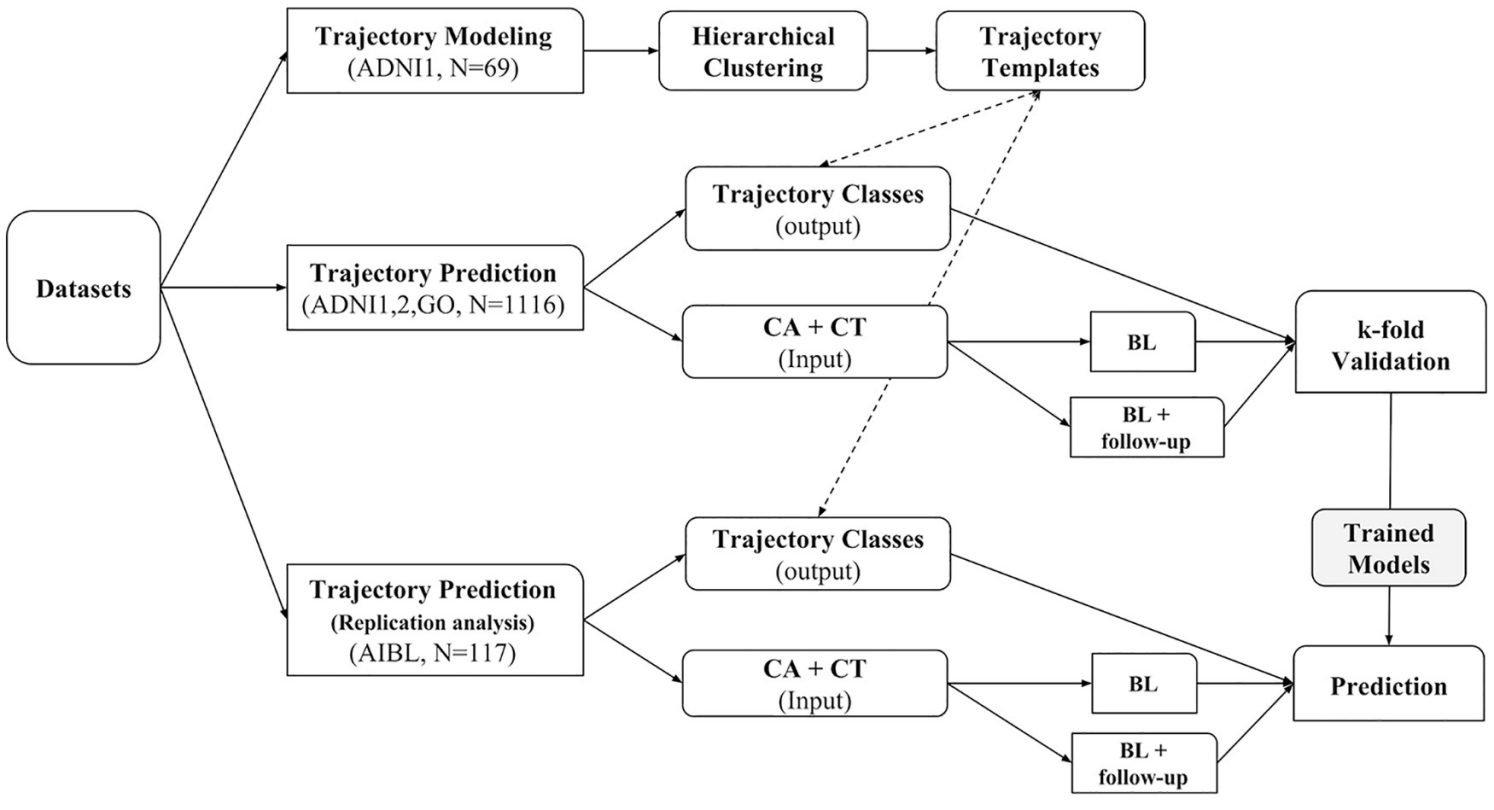
Analysis workflow of the longitudinal framework. The workflow comprises two tasks, 1) trajectory modeling (TM), and 2) trajectory prediction (TP). Data from 69 ADNI-1 MCI subjects with 9 visits within 6 years are used for TM task using hierarchical clustering. 1116 ADNI subjects pooled from ADNI1, ADNIGO, and ADNI2 cohorts are used towards TP task. Data (CA: clinical attributes, CT: cortical thickness) from baseline and a follow-up timepoint is used towards trajectory prediction. The trained models from k-fold cross validation of ADNI subjects are then tested on 117 AIBL subjects as part of the replication analysis.

The presented longitudinal framework comprises two tasks, 1) trajectory modeling and 2) trajectory prediction. The overall process is outlined in Fig 1.

### Trajectory modeling

**Fig 2:**
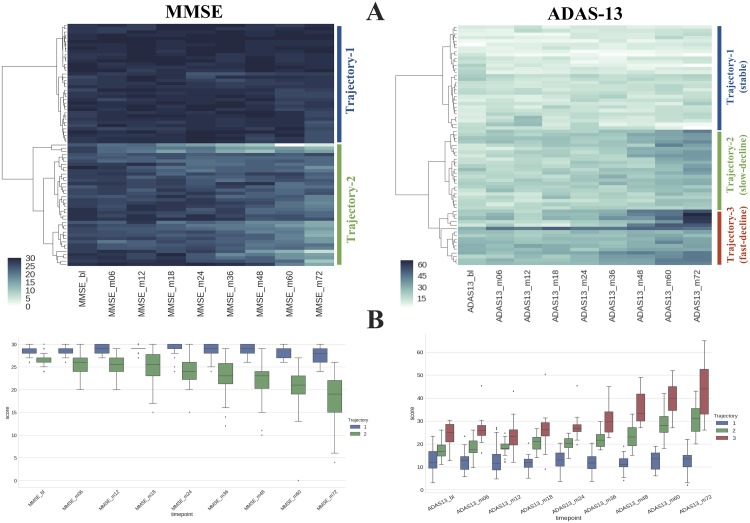
Trajectory modeling. **A)** 69 MCI subjects (rows) with six years of clinical scores (columns) were used as input to hierarchical clustering. The color indicates the clinical score at a given timepoint. Euclidean distance between score vectors was used as a similarity metric between two subjects. Ward’s method was used as a linkage criterion. **B)** Clinical score distribution at each timepoint of different trajectories (stable vs. decliners) derived from hierarchical clustering. Mean scores at each timepoints are used to build a template for each trajectory class.

We aim to characterize the symptomatic progression of subjects based on clinical scores from multiple timepoints using a data driven approach. In this pursuit, we used 69 late-MCI subjects from ADNI1 cohort (see Table 1) with all 6 years (total 9 timepoints) of available clinical data as input to hierarchical clustering (see Fig 2). The goal here is to group subjects with similar clinical progression in order to build a template of differential trajectories. We note that the primary goal of this exercise is not to discover unknown subtypes of AD progression, but to create trajectory prototypes against which all participants can be compared and assigned a trajectory (prognostic) label. These labels provide a goal for the prediction task, which in traditional settings is defined by diagnosis or change in diagnosis, or even symptom profile at a specific timepoint. We used Euclidean distance between longitudinal clinical score vectors as a similarity metric and Ward’s method as linkage criterion for clustering. Note that the number of clusters is a design choice in this approach, which depends on specificity of the trajectory progression that we desire. Higher number of clusters allows modeling of trajectory progression with higher specificity (e.g. slow vs. fast decline). Clinically, it would be useful have more specific prognosis to prioritize and personalize intervention and treatment options. However, it also increases the difficulty of early prediction. In this work, the choice of 2 vs. 3 clusters is made based on the dynamic score range of the clinical assessment. Each of the resultant clusters represents stable or declining symptom trajectories. We modeled MMSE and ADAS-13 trajectories separately. We note that this does not assume independence between the two scales. This is primarily due to high prevalence of these scales in research and clinic, as well as, to demonstrate the feasibility of clustering approach that allows modeling of >2 trajectories with a more symptom specific clinical assessment such as ADAS-13. After clustering, we simply average the clinical scores of subjects from each cluster at each individual timepoint to determine trajectory-templates for each of the classes (i.e. stable, decline).

Subsequently these trajectory-templates are used to assign trajectory labels to the rest of the subjects (not used in clustering) based on Euclidean proximity computed from all available timepoints of a given subject (see Figure B in S5 File). There are two advantages to this approach. First it allows us to group subjects without having to enforce strict cut-offs for defining boundaries, such as MCI to AD conversion within a certain number of months [6,8,10,42]. Second, it offers a relatively simple way of dealing with missing timepoints, as the trajectory-template can be sampled based on clinical data availability of a given subject. In contrast, prediction tasks that are defined based on specific time window have to exclude subjects with missing data and ignore data from additional timepoints beyond the set cut-off (e.g. late AD converters). We assign trajectory labels to all remaining ADNI and AIBL subjects based on their proximity to each of the trajectory-templates computed from at least 3 timepoints in a timespan longer than one year. The demographics stratified by trajectory labels are shown in Tables 2 and 3. Whereas Table 4 shows the subject membership overlap between MMSE and ADAS-13 based trajectory assignment. These trajectory labels are then used as task outcome (ground truth) in the predictive analysis described next.

**Table 2:** Cluster demographics of ADNI trajectory prediction (TP) cohort based on MMSE.

MMSE	N	Age (years)	APOE4 (n_alleles)	Sex	Dx (at baseline)	Geriatric Depression Scale (at baseline)
**T1 (stable)**	674	72.8	0: 443, 1: 195, 2: 36	M: 371, F: 303	CN: 319, SMC: 23, EMCI: 187, LMCI: 144, AD: 1	Mean: 1.21, Stdev: 1.37
**T2 (decline)**	442	74.8	0: 163, 1: 198, 2: 81	M: 246, F: 196	CN: 8, SMC: 3, EMCI: 43, LMCI: 212, AD: 176	Mean: 1.63, Stdev: 1.39

**Table 3:** Cluster demographics of ADNI trajectory prediction (TP) cohort based on ADAS-13.

ADAS-13	N	Age (years)	APOE4 (n_alleles)	Sex	Dx (at baseline)	Geriatric Depression Scale (at baseline)
**T1 (stable)**	585	72.3	0: 399, 1: 163, 2: 23	M: 308, F: 277	CN: 307, SMC: 25, EMCI: 167, LMCI: 83, AD: 3	Mean: 1.18, Stdev: 1.38
**T2 (slow decline)**	184	74.9	0: 98, 1: 61, 2: 25	M: 122, F: 62	CN: 19, SMC: 1, EMCI: 51, LMCI: 105, AD: 8	Mean: 1.50, Stdev: 1.38
**T3 (fast decline)**	346	74.8	0: 108, 1: 169, 2: 69	M: 186, F: 160	CN: 1, SMC: 0, EMCI: 12, LMCI: 168, AD: 165	Mean:1.66, Stdev: 1.38

**Table 4:** Trajectory membership comparison between MMSE and ADAS-13 scales. Note that MMSE only has single decline trajectory.

Trajectory comparison		ADAS-13		
		Stable	Slow-decline	Fast-decline
**MMSE**	**Stable**	**558**	**107**	**9**
	**Decline**	**27**	**77**	**337**

### Trajectory prediction

The goal of this predictive analysis is to identify the prognostic trajectories of individual subjects as early as possible. In the simplest (and perhaps the most ideal) case, the prediction models use information available from a single timepoint, also referred as the baseline timepoint. However, input from a single timepoint lacks information regarding short-term changes (or rate of change) in neuroanatomy and clinical status, which would potentially be useful for increasing specificity of future predictions of decline. In order to leverage this information, we propose a longitudinal Siamese network (LSN) model that can effectively combine data from two timepoints and improve the predictive performance.

**Fig 3:**
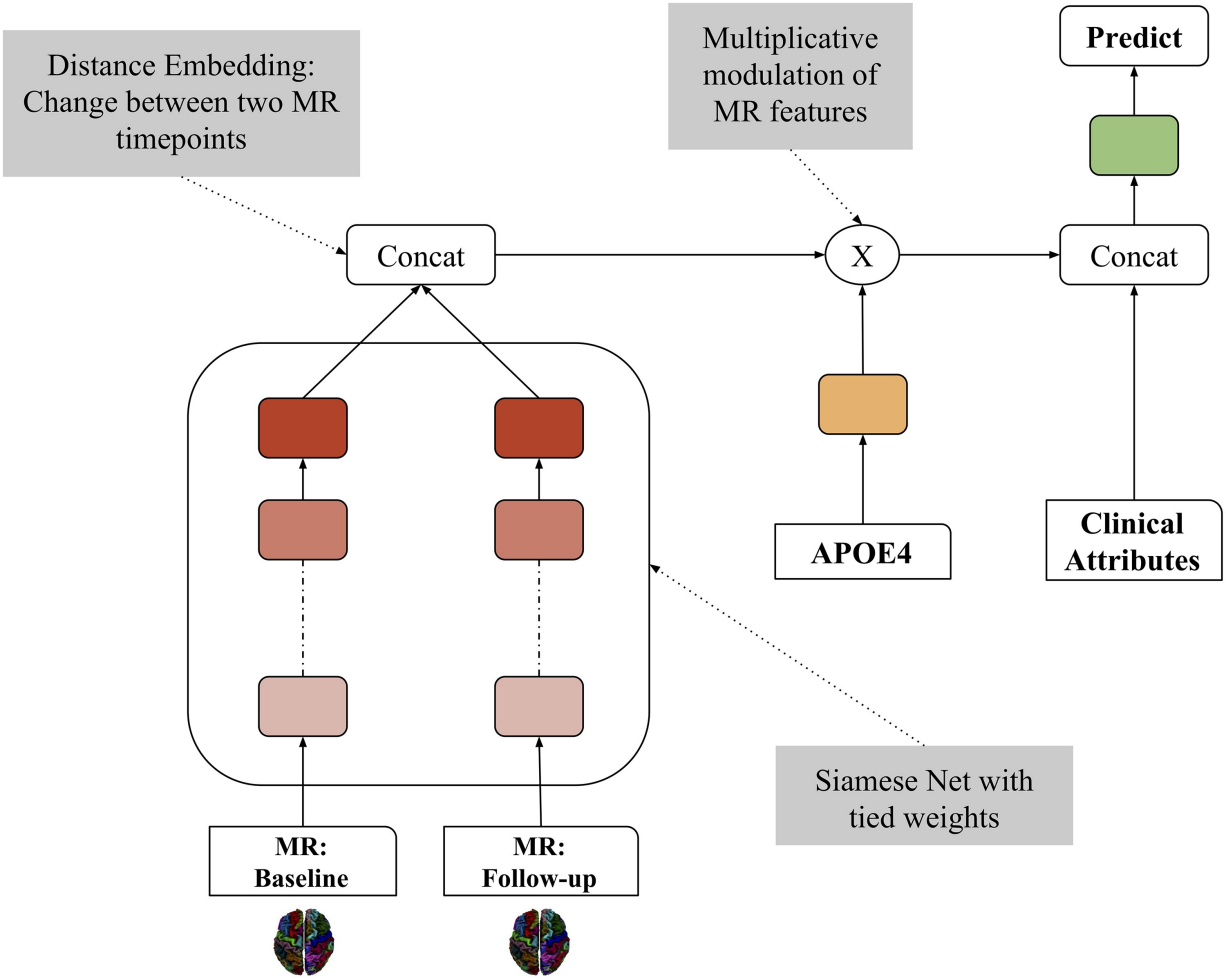
Longitudinal Siamese network (LSN) model. LSN consists of three stages. The first stage is a Siamese artificial neural network with twin weight-sharing branches. Weight-sharing implies identical weight configuration at each layer across two branches. These branches process an MR input (2 x 78 CT values) from the same subject at two timepoints and produce a transformed output that is representative of change (atrophy) over time. This change pattern is referred as “distance embedding”. In the second stage, this embedding is modulated by the APOE4 status with a multiplicative operation. Lastly, in the third stage, the modulated distance embedding is concatenated with the two clinical scores and age, and used towards final trajectory prediction. The weights (model parameters) of all operations are learned jointly in a single unified model framework.

We build the LSN model with two design objectives. First, we want to combine the MR information from two timepoints that encodes the structural changes predictive of different clinical trajectories. Second, we want to incorporate genetic (APOE4 status) and clinical information with the structural MR information in an effective manner. We achieve these objectives with an augmented artificial neural network architecture (see Fig 3) comprising a Siamese network and a multiplicative module, that combines MR, genetic and clinical data systematically.

The first stage of LSN is an artificial neural network model based on a Siamese architecture with twin weight-sharing branches. Typically, Siamese networks are employed for tasks, such as signature or face verification, that require learning a “similarity” measure between two comparable inputs [43,44]. Here, we utilize this architecture towards encoding a “difference embedding” between two MR images in order to represent the amount of atrophy over time that is predictive of clinical trajectory. In the second stage, this encoding is modulated by the APOE4 status with a multiplicative operation. The choice of multiplicative operation is based on an underlying assumption of non-additive interaction between MR and genetic modalities. Subsequently, the modulated distance embedding is concatenated with the baseline and follow-up clinical scores and age, which is used towards final trajectory prediction. The weights (model parameters) of all operations are learned jointly in a single unified model framework. The complete design specification of the model are outlined in Table 5. The complete input data consists of 2 x 78 CT values, 2 x clinical score, age, and APOE4 status. Two different LSN models are trained separately to perform binary classification task for MMSE based trajectories and three-way classification task for ADAS-13 based trajectories. All models are trained using the TensorFlow library (version: 0.12.1, https://www.tensorflow.org/), and code is available at (https://github.com/CobraLab/LSN).

**Table 5:** Longitudinal Siamese network (LSN) architecture.

Network module	Specifications
**Siamese net**	Input: 2 x 78 CT values per subject Number of hidden layers: 4 each branch (fixed for all folds) Number of nodes per layer: 25 to 50 (grid search) Output: distance embedding Number of nodes: 10 to 20 (grid search)
**Multiplicative modulation**	Input: distance embedding, APOE4 status (0, 1, 2) Number of hidden layers: 1 (fixed for all folds) Number of nodes: 1 (fixed for all folds) Output: modulated distance embedding
**Concatenation and prediction**	Input: modulated distance embedding, clinical scores, age Number of hidden layers: 1 (fixed for all folds) Number of nodes: 10 to 20 (grid search) Output: trajectory class

### Selection of second-timepoint as input

LSN model is agnostic to the time difference between two MR scans. Therefore, we use the latest available datapoint for a given subject within 1 year from the baseline visit. This allows us to include subjects with missing data at the 12-month timepoint, but have available MR and clinical data at the 6-month timepoint (N = 43). We note that this is an “input” timepoint selection criterion, as the ground truth trajectory labels (output) are assigned based on all available timepoints (3 or more) for each subject.

### Performance gains from MR modality and the second timepoint

**Fig 4:**
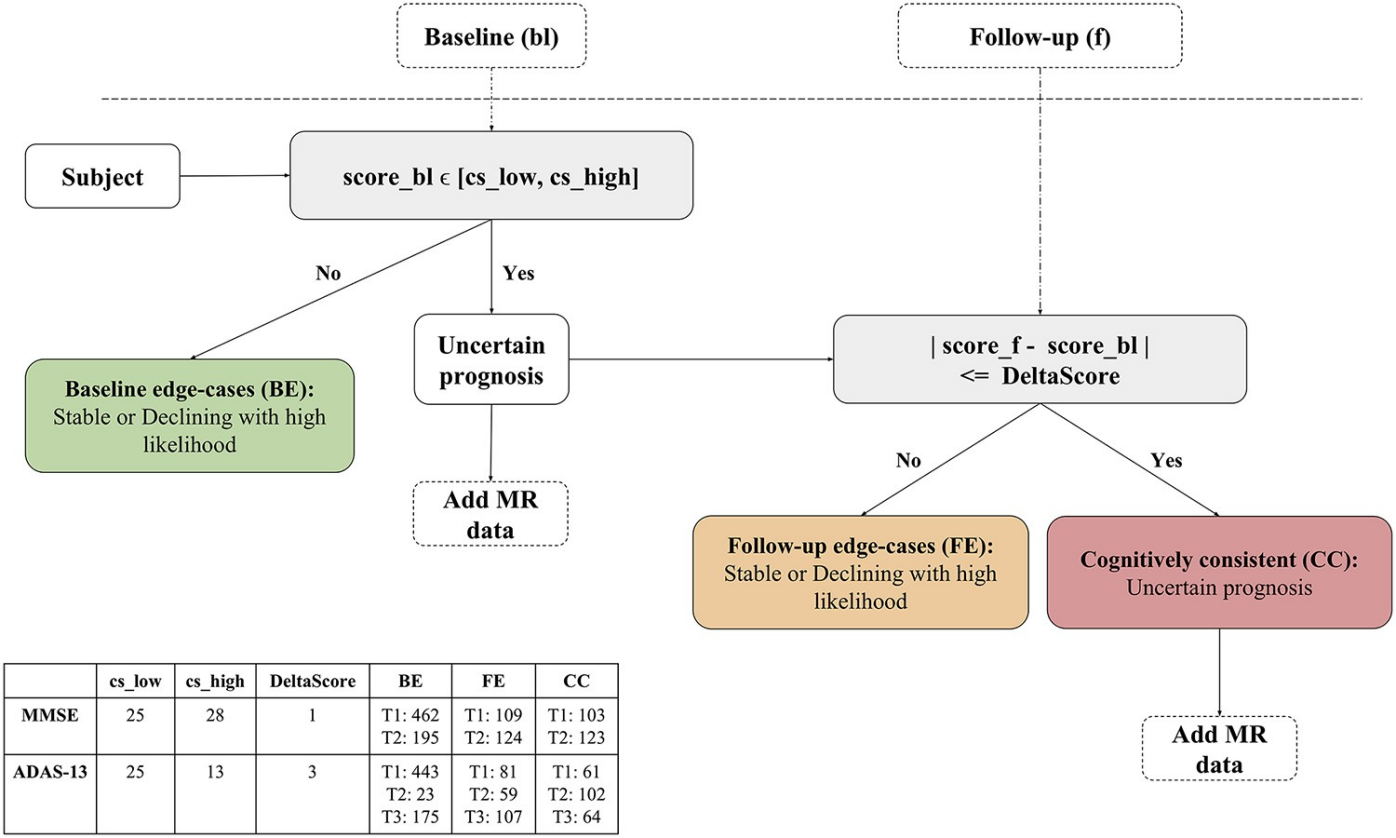
Potential clinical workflow for subject specific decision-making. The goal of this flowchart is to identify subjects benefitting from MR and additional timepoint information. Qualitatively, baseline edge-cases (BE) group includes subjects with very high or very low cognitive performance at baseline. Follow-up edge-cases (FE) group includes subjects with non-extreme cognitive performance at baseline but substantial change in performance at follow-up. And cognitively consistent (CC) group includes subjects with non-extreme cognitive performance at baseline and marginal change in performance at follow-up. The table shows the threshold values used for MMSE and ADAS-13 scales and corresponding trajectory class distribution within each group.

In order to gain insight into identifying subjects that would benefit from added MR and second timepoint information, we divide the subjects into three groups based on a potential clinical workflow as shown in Fig 4.

- Baseline edge-cases (BE): Subjects with very high or very low cognitive performance at baseline
- Follow-up edge-cases (FE): Subjects with non-extreme cognitive performance at baseline but marked change in performance at follow-up
- Cognitively consistent (CC): Subjects with non-extreme cognitive performance at baseline and marginal change in performance at follow-up

The cutoff thresholds for the quantitative stratification, and the corresponding subject-group memberships are shown in Fig 4. We note that these threshold values are not a fixed choice, and are selected here only for the purpose of demonstrating a clinical use case. Based on this grouping, we can expect that the clinical scores of CC subjects provide very little information regarding potential trajectories and thus we need to rely on MR information for prediction, making it the target group for multimodal, longitudinal prediction.

### Performance evaluation

We computed the performance of 2x3 input choices based on two timepoints: 1) baseline, 2) baseline+follow-up and three feature sets: 1) clinical attributes (CA) comprising clinical score, age and apoe4 status, 2) cortical thickness (CT) data comprising 78 ROIs, and 3) combination of CA and CT features. This allows us to evaluate the benefits of added MR-derived information and the second timepoint. Evaluating the added benefit of the MR modality is critical due to the implicit dependency between clinical scores and outcome variables commonly encountered during prognostic predictions. Accuracy, area under the curve (AUC), confusion matrix (CM), and receiver operating characteristic (ROC) were used as evaluation metrics.

All models were evaluated using pooled ADNI1, ADNI2, and ADNIGO subjects in a 10-fold nested cross-validation paradigm (see S6 File). By pooling subjects from ADNI1, ADNIGO, and ADNI2 we show that the models trained in this work are not sensitive to differences in acquisition and quality of the MR data (e.g. 1.5T vs. 3T). Hyper-parameter tuning was performed using nested inner folds. The ranges for hyperparameter grid search are provided in S2 File. The train and test subsets were stratified by cohort membership and the trajectory class distribution. The stratification minimizes the risk of overfitting models towards particular cohort and reduces cohort specific biases during evaluation of results.

We compare the performance of LSN with four reference models: logistic regression with Lasso regularizer (LR), support vector machine (SVM), random forest (RF), and default artificial neural network (ANN). The comparison of LSN against other models allows us to quantify the performance gains offered by LSN with data from two timepoints. We hypothesize that LSN would provide better predictive performance over existing prediction modeling approaches comprising single as well as two timepoints. For reference models, all input features under consideration were concatenated into a single array. For LR and SVM models, all features in a given training set were standardized by removing the mean and scaling to unit variance. The test set was standardized using the mean and standard deviation computed from the training set. The statistical comparison of LSN against other models was performed using Mann-Whitney-U test. All trained models were later directly used to test predictive performance with AIBL subjects. The AIBL performance statistics were averaged over 10 instances of trained models (one from each fold) for each input combination.

## Results

### Trajectory modeling

MMSE and ADAS-13 based clustering yielded two and three clusters, respectively. The bigger score range of ADAS-13 scale (see Figure A in S3 File) allows modeling of symptom progression with higher specificity providing slow and fast decline trajectories. The cluster assignment of the entire ADNI dataset based on the trajectory-templates yielded 674 stable and 442 decline subjects for MMSE scale, and 585 stable, 184 slow decline, and 346 fast decline subjects for ADAS-13 scale. The complete cluster demographics are shown in Tables 2 and 3. Notably, stable clusters comprise higher percentage subjects with zero copies of APOE4 allele, and conversely declining clusters comprise higher percentage of subjects with two copies of APOE4 allele. Subjects with single APOE4 copy are evenly distributed across stable and declining clusters. Table 4 shows the trajectory membership overlap between MMSE and ADAS-13. The results indicate that large number of subjects belong to similar trajectories for MMSE and ADAS-13. In the non-overlapping cases, especially the stable MMSE subjects predominantly belong to slow-decline ADAS-13 trajectory.

### Trajectory prediction

**Fig 5:**
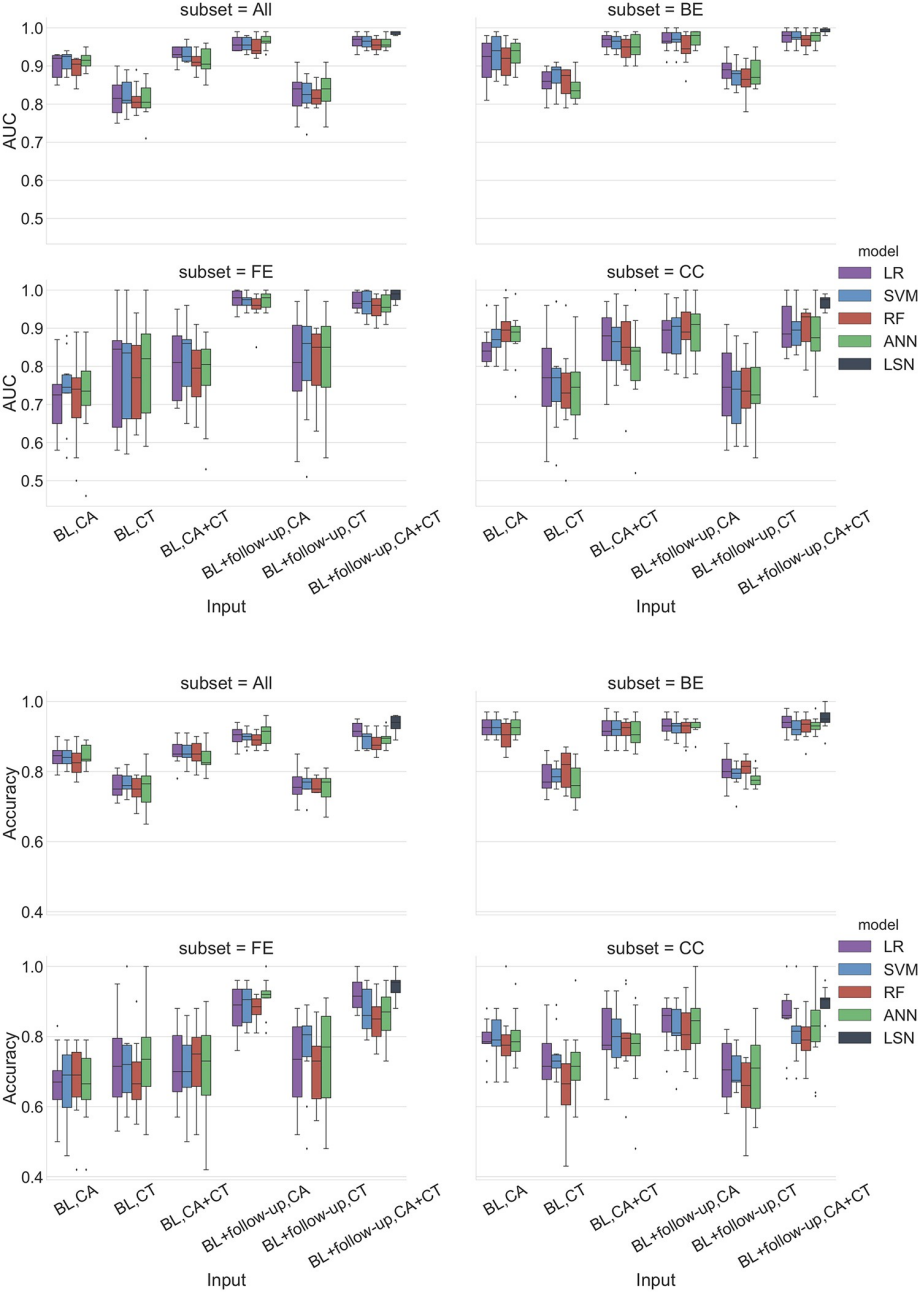
MMSE prediction AUC and accuracy performance for ADNI dataset. Top panel: Area under the ROC curve (AUC), Bottom panel: Accuracy. Results are stratified by groups defined by the clinical workflow. Abbreviations are as follows. BL: baseline visit, CA: clinical attributes, CT: cortical thickness. BE: baseline edge-cases, FE: follow-up edge-cases, CC: cognitively consistent. The statistical comparison of LSN against other models was performed using Mann-Whitney-U test. Note that only BL+followup, CA+CT input is relevant for this comparison. For AUC comparison, LSN offered significantly better performance over all four models for ‘All’, ‘BE’, and ‘CC’ subsets. LSN also offered statistically significant results over RF and ANN models for ‘FE’ subset. For accuracy comparison, LSN offered significantly better performance over SVM, RF, and ANN models for ‘All’, ‘FE’, and ‘CC’ subsets. No statistically significant results were obtained for ‘BE’ subset.

**Fig 6:**
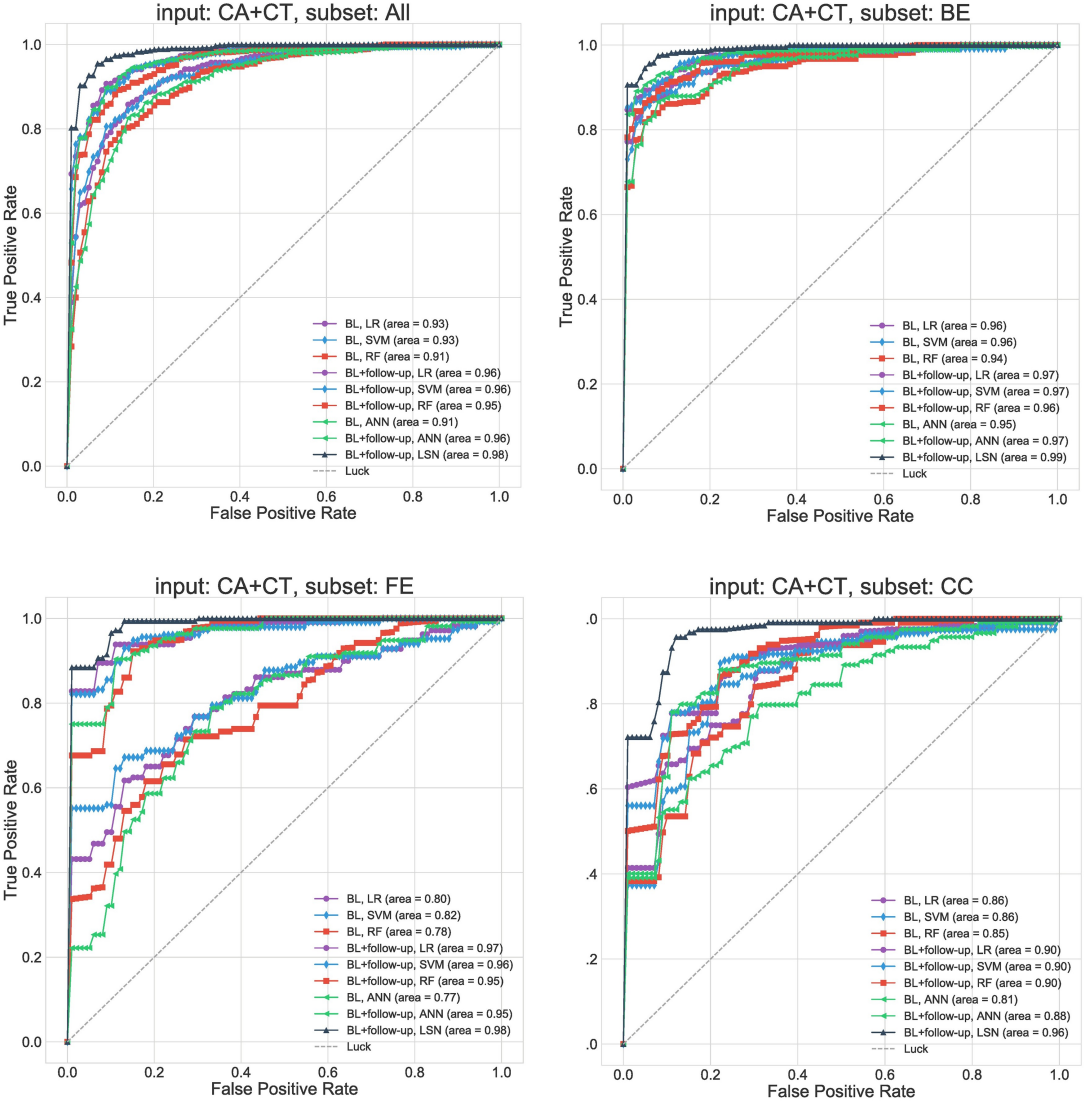
MMSE prediction ROC curves for ADNI dataset. **Receiver operating characteristic curves for CA+CT input**. Results are stratified by groups defined by the clinical workflow. Abbreviations are as follows. BL: baseline visit, CA: clinical attributes, CT: cortical thickness. BE: baseline edge-cases, FE: follow-up edge-cases, CC: cognitively consistent.

**Fig 7:**
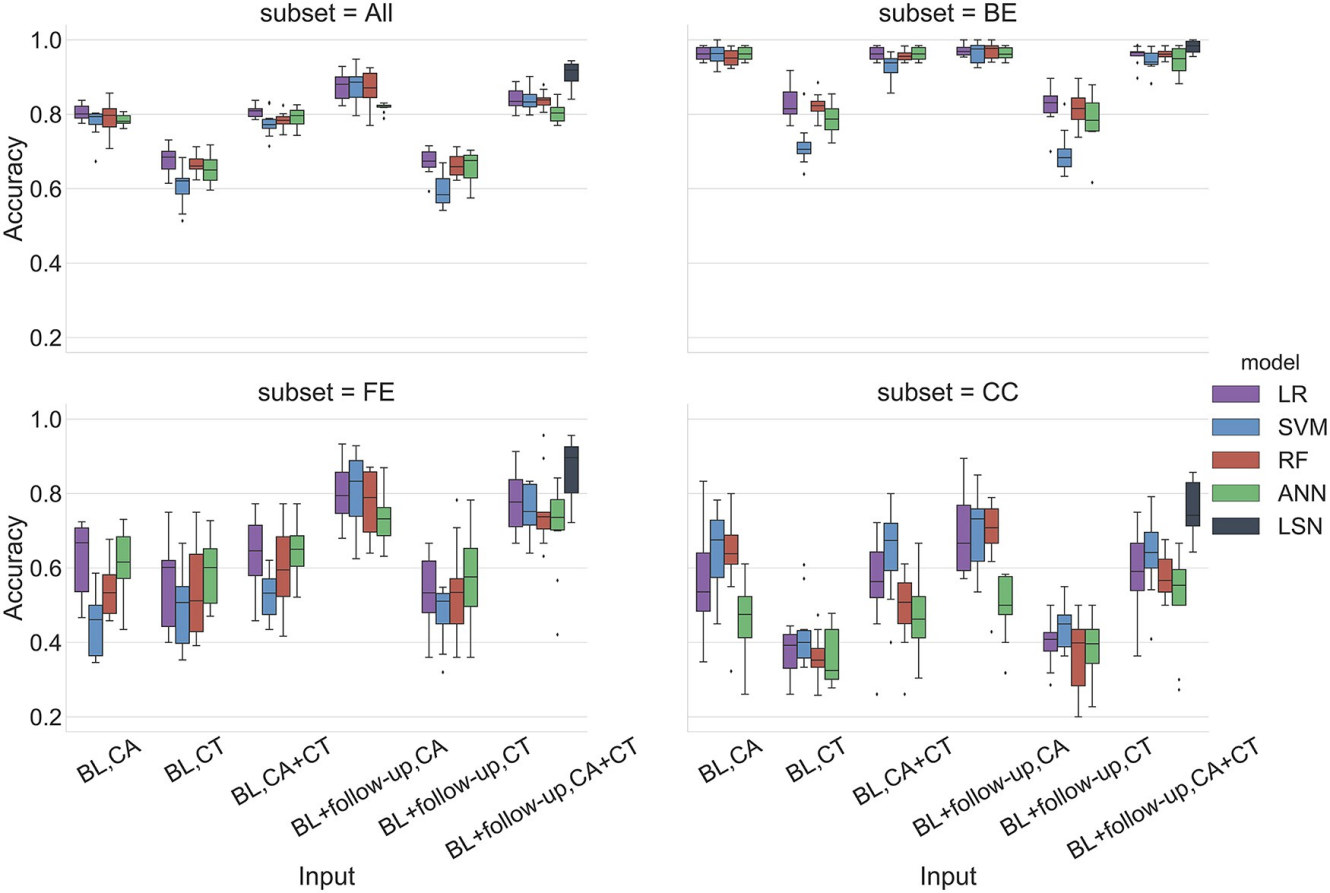
ADAS-13 prediction accuracy performance for ADNI dataset. Results are stratified by groups defined by the clinical workflow. Abbreviations are as follows. BL: baseline visit, CA: clinical attributes, CT: cortical thickness. BE: baseline edge-cases, FE: follow-up edge-cases, CC: cognitively consistent. The statistical comparison of LSN against other models was performed using Mann-Whitney-U test. Note that only BL+followup, CA+CT input is relevant for this comparison. For accuracy comparison, LSN offered significantly better performance over LR, SVM, RF, and ANN models for ‘All’, ‘FE’, ‘BE’, and ‘CC’ subsets.

**Fig 8:**
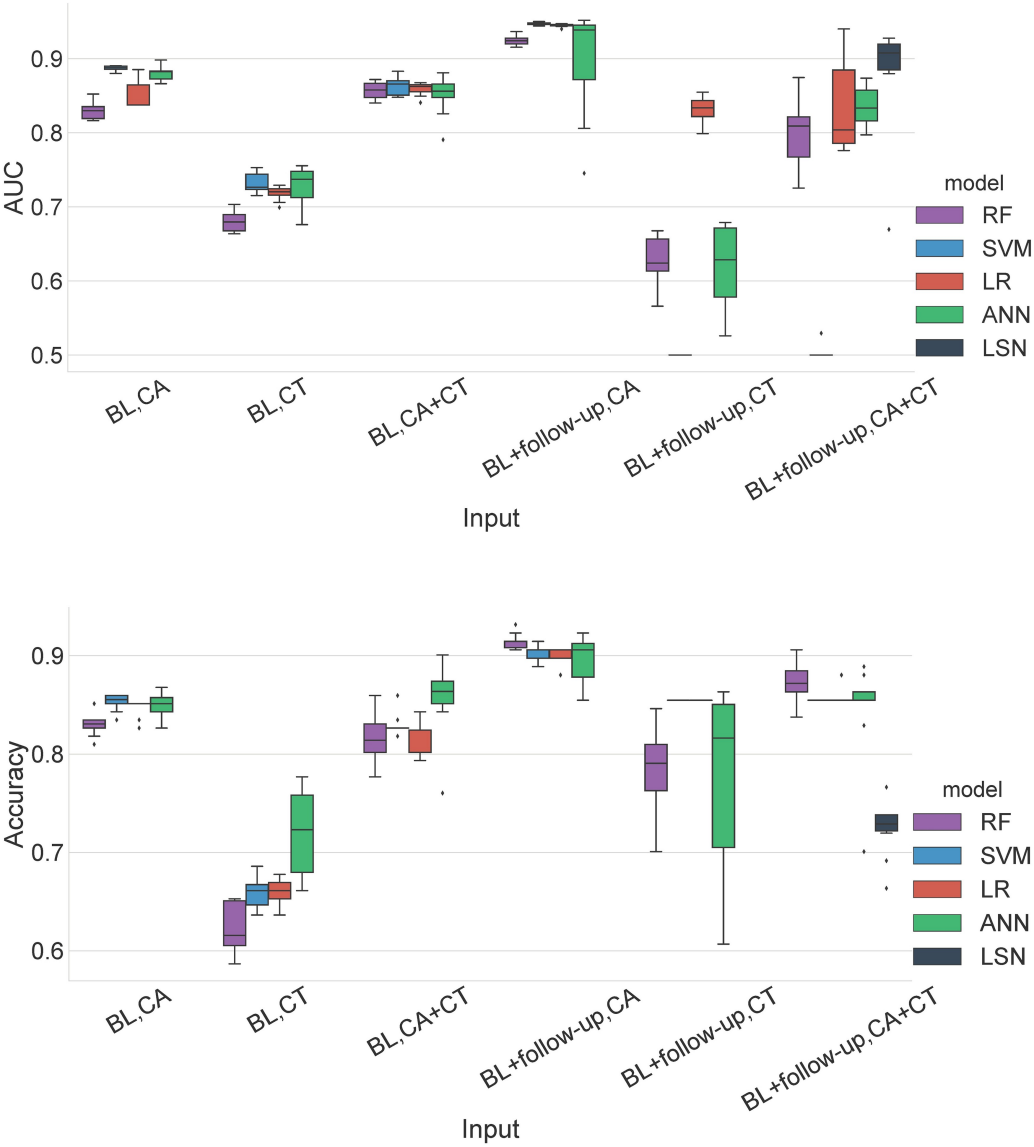
MMSE prediction AUC and accuracy performance for AIBL replication cohort. Top pane: Area under the ROC curve (AUC), Bottom pane: Accuracy. Abbreviations are as follows. BL: baseline visit, CA: clinical attributes, CT: cortical thickness.

**Fig 9:**
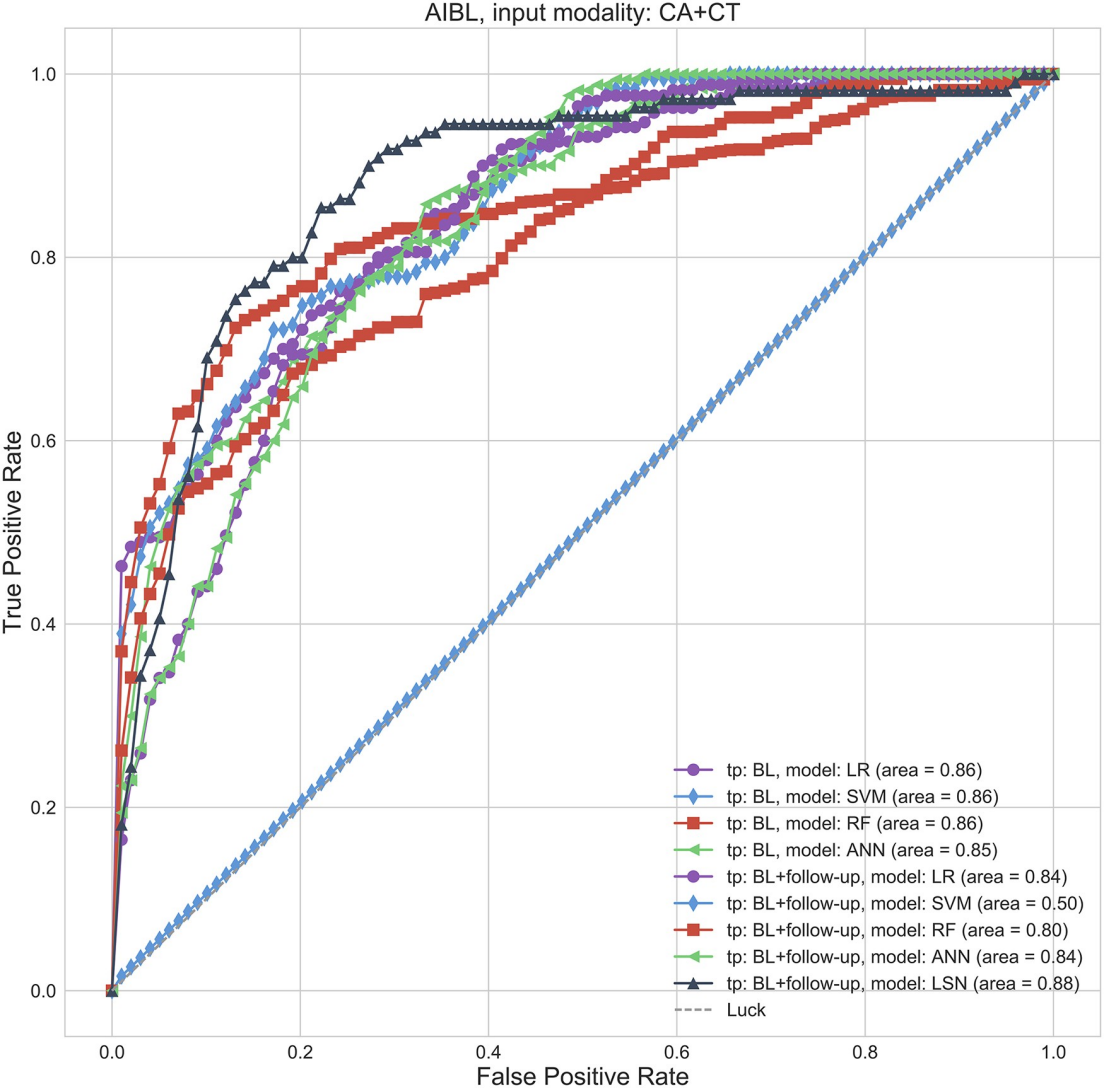
MMSE prediction ROC curves for AIBL replication cohort. Receiver operating characteristic curves for CA+CT input. Abbreviations are as follows. BL: baseline visit, CA: clinical attributes, CT: cortical thickness.

Below we provide the MMSE and ADAS-13 based trajectories prediction results for single (baseline) and two timepoint (baseline + follow-up) models for 1) CA 2) CT, and 3) CA+CT input. Results are summarized in Figs 5–9 and Tables A-G in S4 File. Note that ADAS-13 based trajectory results are from a 3-class prediction task. We first report the performance LSN model, which is only applicable to the two timepoint input comprising combined CA and CT features, followed by performance of single and two timepoint reference models.

### MMSE trajectories (binary classification)

The combined CA and CT input feature set with two timepoints provides the most accurate prediction of trajectory classes. LSN outperforms all four reference models with 0.94 accuracy and 0.99 AUC (see Figs 5 and 6, Table C in S4 File) with all subjects, and 0.900 accuracy and 0.968 AUC for “cognitively consistent” group.

### Single (baseline) timepoint models

With only CA input, all four reference models (LR, SVM, RF, ANN) provide highly similar performance with top accuracy of 0.84 and AUC of 0.91 (see Figs 5 and 6, Tables A-C in S4 File). With CT input, the performance diminishes for all models with top accuracy of 0.77 and AUC of 0.83. With combined CA+CT input, all four models see a boost with top accuracy of 0.86 and AUC of 0.93.

### Two timepoints (baseline + second-timepoint)

With only CA input, all four models (LR, SVM, RF, ANN) provide highly similar performance with top accuracy of 0.91 and AUC of 0.97 (see Figs 5 and 6, Tables A-C in S4 File). With CT input, the performance diminishes for all models with top accuracy of 0.77 and AUC of 0.83. With combined CA+CT input, the performance stays similar to CA input for all four models with top accuracy of 0.91 and AUC of 0.96.

### Clinical group trends

The groupwise results (see Figs 5 and 6, Tables A-C in S4 File) show that the trajectories of “baseline edge-cases (BE)” can be predicted with high accuracy by their baseline clinical attributes. Consequently, inclusion of CT and a second timepoint features offers minimal gains to predictive accuracy. Nevertheless, the two-timepoint LSN model with CA and CT inputs does offer the best performance with 0.95 accuracy and 0.992 AUC. Interestingly, trajectory prediction of “follow-up edgecase (FE)” yields poor performance with baseline CA features. Inclusion of baseline CT features improves performance for the FE group. Higher predictive gains are seen after inclusion of second timepoint information, with LSN model offering the best performance with 0.942 accuracy and 0.987 AUC. Lastly, trajectory prediction of “cognitively consistent (CC)” subjects, shows in between performance compared to BE and FE subjects with baseline data. However, inclusion of second timepoint does not improve performance substantially, causing poorer prediction of CC subjects compared to FE and BE with two-timepoints, with LSN still offering the best performance.

### ADAS-13 trajectories (3-way classification)

Similar to MMSE task, the combined CA and CT feature set with two timepoints provides the most accurate prediction of trajectory classes. LSN outperforms all four reference models with 0.91 accuracy (see Fig 7, Table F in S4 File) with all subjects, and 0.76 accuracy for “cognitively consistent” group.

### Single (baseline) timepoint (all subjects)

With only CA input, LR provides the top accuracy of 0.81. The confusion matrix (CM) shows that all four models perform relatively better at distinguishing subjects in stable and fast-declining trajectories, compared to identifying slow-declining trajectory from the other two (see Fig 7, Tables D-F in S4 File). The ANN model does not offer consistent performance for slow-declining trajectory prediction as it fails to predict that class in at least one fold of cross validation. With CT input only, the performance diminishes for all models with top accuracy of 0.68 with LR model. The issue regarding the slow-declining trajectory prediction is persistent with CT features as both LR and RF models fail to predict that class in at least one fold of cross validation. With combined CA+CT input, the performance remains similar to CA input for all four models with top accuracy of 0.81.

### Two timepoints (baseline + second-timepoint) (all subjects)

With only CA input, LR and SVM provide similar performance with accuracy of 0.88. Similar to single timepoint models, the confusion matrix (CM) shows that all four models perform relatively better at distinguishing subjects in stable and fast-declining trajectories, compared to identifying slow-declining trajectory from the other two (see Fig 7, Tables D-F in S4 File). With CT input only, the performance diminishes for all models with top accuracy of 0.67 with LR model. Both LR and RF models fail to predict slow-declining trajectory with CT features in at least one-fold of cross validation. With combined CA+CT input, the performance diminishes compared to CA input for all four models with top accuracy of 0.84.

### Clinical group trends

The groupwise results (see Fig 7, Tables D-F in S4 File) show that the trajectories of “baseline edge-cases (BE)” can be predicted with high accuracy by their baseline clinical attributes. Consequently, inclusion of CT and the second timepoint features offers minimal gains to predictive accuracy. The confusion matrix shows that only stable and fast declining trajectories can be predicted due to extreme scores of BE subjects. The two-timepoint LSN model with CA and CT features offers the best performance with 0.98 accuracy. Trajectory prediction of “follow-up edgecase (FE)” yields poor performance with baseline CA features. Inclusion of baseline CT features only incrementally improves performance. Substantial predictive gains are seen after inclusion of second timepoint information, with LSN model offering the best performance with 0.86 accuracy. The confusion matrix shows predictability of all three classes. Lastly, trajectory prediction of “cognitively consistent (CC)” subjects shows in between performance with baseline data compared to BE and FE. However, inclusion of a second timepoint does not improve performance substantially, causing poorer prediction of CC subjects compared to FE and BE with two-timepoints. Nevertheless, LSN still offers the best performance compared to the reference models.

### AIBL results

The use of AIBL as a replication cohort has a few caveats due to differences in the study design and data collection. Notably, AIBL has an 18-month interval between timepoints with at most 4 timepoints per subject and does not have ADAS-13 assessments available. Therefore, the results are provided only with MMSE assessments. The trajectory membership assignment based on ADNI templates, yielded 99 stable and 18 declining subjects in the AIBL cohort. For MMSE based trajectory classification task, LSN offers 0.724 accuracy and 0.883 AUC with combined CA and CT feature set from two timepoints.

### Single (baseline) timepoint (all subjects)

With only CA input, LR, SVM, RF provide similar performance with SVM offering the best results with 0.85 accuracy and 0.88 AUC. With only CT input, performance of all models degrades substantially with maximum of 0.72 accuracy and 0.74 AUC. With combined CA+CT input, performance of reference models is similar to CA input, with ANN offering the best performance with 0.85 accuracy and 0.85 AUC values (see Figs 8 and 9, Table G in S4 File).

### Two timepoints (baseline + second-timepoint) (all subjects)

With only CA input, LR, SVM, RF provide similar performance with LR and SVM offering the best results with 0.90 accuracy and 0.95 AUC. With only CT input, performance of all models degrades, and especially LR and SVM offer biased performance with at least one model instance predicting only the majority class (i.e. stable). With combined CA+CT input, again LR, SVM, and ANN offer biased performance with at least one model instance predicting only the majority class (i.e. stable). RFC offers relatively balanced performance with 0.87 accuracy and 0.80 AUC (see Figs 8 and 9, Table G in S4 File).

### Effect of trajectory modeling on predictive performance

The use of variable number of clinical timepoints per subject based on the availability during trajectory assignment (groundtruth) impacts the predictive performance. The stratification of performance based on last available timepoint for a given subject yielded 605, 510 subjects for 18 to 36 months (near future) and 48 to 72 month (distant future) spans, respectively. The AUC results for MMSE based trajectories show (see Figure B in S5 File) that all models perform better while predicting trajectories for subjects with visits from shorter timespan (up to 36 months). The predictive performance worsens for subjects with available timepoints between 48 and 72 months. This difference in the performance is largest with CA input and lowest with CA+CT input. The 3-way accuracy results for ADAS-13 based trajectories (see Figure C in S5 File) show a similar trend with CA input as all models perform better for subjects with visits from shorter time span. However with CT and CA+CT input, the amount of bias varies with the choice of model. While comparing models, LSN shows the least amount of predictive bias subjected to timepoint availability for both prediction tasks (MMSE and ADAS-13).

### Computational specifications and training times

The computation was performed on Intel(R) Xeon(R) CPU E5-1660 v3 @ 3.00GHz machine with 16 cores and a GeForce GTX TITAN X graphic card. Scikit-learn (http://scikit-learn.org/stable/) and TensorFlow (version: 0.12.1, https://www.tensorflow.org/) libraries were used to implement and evaluate machine-learning models. The training times can vary based on hyperparameter grid search. The average training times for a single model are as follows: 10 minutes for LSN, 3 minutes for ANN, and less than a minute for LR, SVM, and RF.

## Discussion

In this manuscript, we aimed towards building a framework for longitudinal analysis comprising modeling and prediction tasks that can be used towards accurate clinical prognosis in AD. We proposed a novel data-driven method to model clinical trajectories based on clustering of clinical scores measuring cognitive performance, which were then used to assign prognostic labels to the individuals. Subsequently, we demonstrated that longitudinal clinical and structural MR imaging data along with genetic information can be successfully combined to predict these trajectories using machine-learning techniques. Below, we highlight the clinical implications of the proposed work, followed by the discussion of trajectory modeling, prediction, replication task, comparisons to related work in the field, and the limitations.

### Clinical implications

A critical component of any computational work seeking to predict patient outcome is its clinical relevance. How can the proposed methodology be integrated into a clinical workflow? Is the use of expensive and potentially stressful tests worthwhile in a clinical context? How can we use *change* in clinical status and neuroanatomical progression in a short-term to better predict long-term outcomes for patient populations? We envision two overarching clinical uses for the presented work. First our trajectory modeling efforts provide a symptom-centric prognostic objective across AD and prodromal population. Then the prediction model facilitates decision-making pertaining to frequency and types of assessments that should be conducted in preclinical and prodromal individuals. If an individual is predicted to be in fast-decline, then this may lead a clinician to recommend more frequent assessments, additional MRI sessions, and preventative therapeutic interventions. Conversely, if an individual is deemed as being stable (in spite of an MCI diagnosis) this may lead a clinician to reduce the frequency of assessments. Another application would be assisting clinical trial recruitment based on the projected rate of decline. Pre-selection of individuals for clinical trials has become an important topic in AD given the recent challenges in the drug development [45–47]. LSN can help identify the suitable candidates who are at high risk for decline over the next few years.

The workflow shown in Fig 4 provides a use case for motivating continual monitoring and adopting LSN in the clinic. We note that the stratification of clinical spectrum is not a trivial problem [18], and is confounded by several demographic and genetic factors. Nonetheless, the example clinical scenario in Fig 4 may help identification of specific individuals whose prognosis may be improved by acquiring additional data (such MRI or a follow-up time point). The results (see Figs 5 and 7) suggest that prognosis of subjects on the extreme ends of the spectrum i.e. baseline edge-cases (BE), can be simply predicted by their CA features. However, same features yield poor performance for follow-up edge-cases (FE) subjects, which exhibit substantial change in clinical performance in the one year period from baseline. This shows that models using baseline CA inputs simply predict continuation of symptom severity as seen at baseline. Inclusion of CT features does help compensate some of this prediction bias as seen by improved performance with the CA+CT input at baseline. Lastly cognitively consistent (CC) subjects, which perform in the mid-ranges of clinical performance at baseline and do not show substantial change within a year, are predicted poorly with CA input at baseline and with the inclusion of second timepoint. This is expected since unlike the BE and FE groups, clinical scores from two timepoints for CC group do not point towards one trajectory or the other. Inclusion of MR features from both timepoints seems to be highly beneficial for these subjects, especially with LSN model. These observations demonstrate the utility of short-term longitudinal MR input towards long-term clinical prediction.

### Trajectory modeling

Of importance to our modeling work are the challenges addressed in the longitudinal clinical task definition. The modeling approach defines stable and declining trajectories over six years without the need for hard thresholds (e.g. time window, four-point change etc.). Moreover, it also offers a solution to deal with subjects with missing timepoints. The hierarchical clustering approach provides control over desired levels of trajectory specificity. The higher memory related specificity of ADAS-13 coupled with the larger score ranges allows us to resolve three trajectories (clusters), with further subdivision of decline trajectory. The trajectory membership comparison between MMSE and ADAS-13 scales (see Table 4) shows that there is large number of subjects that overlap in the stable and fast declining group. This is expected due to several commonalities between the two assessments. The non-overlapping cases could be resultant of assessments of specific symptom subdomain performance and higher specificity offered by the three ADAS-13 trajectories over two MMSE trajectories.

### Trajectory prediction

Pertaining to prognostic performance, it is imperative to discuss 1) prediction accuracy versus trajectory specificity trade-off and 2) prediction gains offered by the additional information (MR modality, follow-up timepoint). The comparison between MMSE and ADAS-13 prediction confirms that the choice of three trajectories for ADAS-13 offers more prognostic specificity, with lower mean accuracy for the 3-way prediction. While evaluating longitudinal clinical prediction, it is important to note the implicit dependency between the task outcomes (e.g. future clinical score prediction, diagnostic conversion etc.) and baseline clinical scores. Seldomly do studies disambiguate between what can be achieved in longitudinal prediction tasks using only baseline clinical scores. Our results on the entire cohort (Figs 5 and 7, subset = All) suggests that at baseline, for large number of subjects, high prediction accuracy can be achieved solely with clinical inputs with incremental gains from CT inputs. Interestingly, follow-up clinical information improves predictions over multimodal performance at baseline. Lastly, the multimodal, two-timepoint input offers the best performance compared to all other inputs with LSN, underscoring the importance of model architecture for multimodal, longitudinal input. We attribute this performance to the design novelties that allow the model to learn feature embeddings that are more predictive of the clinical task compared to models that simply concatenate all the features into a single vector, as is often done [13,48]. Siamese network with shared weights provides an effective way for combining temporally associated multivariate input to represent change patterns using two CT feature sets from the same individual. Subsequently, LSN modulates the anatomical change by APOE4 status and combines with clinical scores. We also note that LSN can be extended to incorporate high dimensional voxel or vertex-wise input as well; however, considering the performance offered by ROI based feature selection approach, we estimate marginal improvements at substantially higher computational costs.

### AIBL analysis

The goal of AIBL analysis was to go beyond standard K-fold cross validation and evaluate model robustness with subjects from an independent study that was not used to train the model. The differences in the study design and data collection such as the 18 month interval between timepoints and the last visit at 54 month mark, do introduce a few issues. First, although the flexibility of trajectory modeling approach allows trajectory membership assignment with any number of available timepoints, the shorter study span introduces bias compared to ADNI subjects with typically more number of available timepoints spread over larger timespan. Consequently, we see a highly skewed trajectory membership assignment with 99 stable and 18 declining subjects. As a result, the accuracy and confusion matrix results show that references models are skewed towards prediction of majority class with baseline and two timepoint. Especially with inclusion of CT features in two-timepoint LR and SVM models seems to predict only majority class. This can be potentially attributed to the feature standardization issue. Different datasets have different MR feature distribution, which can make application of scaling factors learned on one dataset (ADNI) onto the other dataset (AIBL) problematic—especially for subject level prediction tasks. The prediction with CA input from two timepoints with the 18 month interval are highly accurate, diminishing the need for MR features. This again can be attributed to shorter timespan availability (near future) during the trajectory assignment for the AIBL subjects. We speculate that with the availability of more timepoints (distant future), LSN would see a boost in performance. Nevertheless, from a model stability perspective, LSN offers the best AUC (0.883) with CA+CT input from two timepoints validating its robustness with multi-modal, longitudinal input data types.

### Effect of trajectory modeling on predictive performance

To better understand performance, it is critical to understand the impact of the number of timepoints available for establishing a ground truth classification based on the data that was used. Specifically for MMSE-based predictions (see Figure B in S5 File), the models provide improved performance for individual trajectories over near future (36 months) as opposed to long-term predictions (72 months). There is variable bias shown by models with CT and CA+CT inputs for ADAS-13 based trajectories (see Figure C in S5 File). This could be attributed to the different performance metrics (accuracy vs. AUC) and the 3-way classification task as opposed to the binary MMSE task. LSN shows the least amount of predictive bias offering best results for short-term and long-term predictions for both MMSE and ADAS-13. This could be potentially due to the set of features extracted by the LSN model which captures not only the pronounced short-term markers but also the subtle changes that are indicative of long-term clinical progression.

### Comparison with related work

Past studies [14,15] propose different methods for trajectory modeling and report varying number of trajectories. Authors of [14] construct a two-group quadratic growth mixture model that characterizes the MMSE scores over a six year period. Authors of [15] used a latent class mixture model with quadratic trajectories to model cognitive and psychotic symptoms over 13.5 years. The results indicated presence of 6 trajectory courses. The more popular, diagnostic change-based trajectory models usually define only 2 classes such as AD converters vs. non-converters, or more specifically in the context of MCI population, and stable vs. progressive MCI groups [8,10,42]. The two-group model is computationally convenient; hence the nonparametric modeling approach presented here starts with two trajectories, but can be easily extended to incorporate more trajectories, as shown by 3-class ADAS-13 trajectory definitions.

Pertaining to prediction tasks, it is not trivial to compare LSN performance with previous studies due to differences in task definitions and input data types. Authors of [8,10,42] have provided a comparative overview of studies tackling AD conversion tasks. In these compared studies, the conversion times under consideration range from 18 to 54 months, and the reported AUC from 0.70 to 0.902, with higher performance obtained by studies using combination of MR and cognitive features. The best performing study [8] among these propose a two-step approach in which first MR features are learned via a semi-supervised low density separation technique, which are then combined with cognitive measures using a random forest classifier. Authors report AUC of 0.882 with cognitive measures, 0.799 with MR features, and 0.902 with aggregate data on 264 ADNI1 subjects. Another recent study [9] presents a probabilistic multiple kernel learning (pMKL) classifier with input features comprising variety of risk factors, cognitive and functional assessments, structural MR imaging data, and plasma proteomic data. The authors report AUC of 0.83 with cognitive assessments, 0.76 with MR data, and 0.87 with multi-source data on 259 ADNI1 subjects.

A few studies have also explored longitudinal data towards predictive tasks. The authors of [6] use a sparse linear regression model on imaging data and clinical scores (ADAS and MMSE), with group regularization for longitudinal feature extraction, which is fed into an SVM classifier. The authors report AUC of 0.670 with cognitive scores, 0.697 with MR features, and 0.768 with the proposed longitudinal multimodal classifier on 88 ADNI1 subjects. The authors also report AUC of 0.745 using baseline multimodal data. Another recent study [10] uses a hierarchical classification framework for selecting longitudinal features and report AUC of 0.754 with baseline features and 0.812 with longitudinal features solely derived from MR data from 131 ADNI1 subjects. Despite the differences in task definition, sample sizes, etc., we note a couple of trends. First, as mentioned earlier due to implicit dependency between task definition and cognitive assessments, we see a strong contribution by clinical scores towards the predictive performance with larger cohorts. Then, the longitudinal studies show promising results with performance gains from the added timepoint further motivating models that can handle both multimodal and longitudinal data.

### Limitations

In this work, we used 69 LMCI subjects for trajectory modeling based on availability of complete clinical data. Ideally, this cohort could be expanded in the future to better represent clinical heterogeneity in the at-risk population, as more longitudinal data of subjects across the pre-AD spectrum become available. This would help improve the specificity of the clinical progression patterns used as trajectory templates.

The proposed LSN model is designed to leverage data from two timepoints. Therefore, availability longitudinal CT and CA data along with the APOE4 status is essential for prediction. Although we offer solutions to mitigate issues with missing timepoints (i.e. flexibility of choice for baseline and follow-up visits for LSN), addressing scenarios with missing modality (i.e. APOE4 status) is beyond the scope of this work. We acknowledge that missing data is an important open challenge in the field that affects the sample size of subjects under consideration, making it harder to train and test models. Potentially, one can employ flexible model architectures that can aggregate predictions independently or via Bayesian framework from available modalities. However we defer investigation of these strategies to future work. Then in this work, we compared LSN against standard implementations of the reference model. We note that modification can be made to reference models to incorporate longitudinal input instead of simple concatenation of variables through regularization and feature selection techniques [6,49]. However, such modifications are not trivial, and flexibility offered by the reference models is limited in comparison with ANNs. Lastly, the hierarchical architecture (multiple hidden layers) poses challenges in interpreting the learned features from the CT data. Inferring the most predictive cortical regions from the distance embedding learned by a Siamese network is not a trivial operation. Computationally, the weights in the higher layers that learn robust combinatorial features cannot be uniquely mapped back onto input (i.e. cortical regions). Moreover, from the biological perspective, if there is significant heterogeneity within spatial distribution of atrophy patterns, as observed in MCI and AD, then presence of underlying neuroanatomical subtypes relating to disparate neuroanatomical atrophy patterns is quite plausible. In such scenario, the frequentist ranking of ROI contribution, even if computable, will not be accurate, as it would average the feature importance across subtypes. These limitations are common trade-offs encountered with multivariate, ANN based ML approaches in order to gain more predictive power. We plan to address these issues in future work as suitable techniques become available for neuroimaging data.

### Conclusions

In summary, we presented a longitudinal framework that provides a data-driven, flexible way of modeling and predicting clinical trajectories. We introduced a novel LSN model that combines clinical and MR data from two timepoints and provides state of the art predictive performance. We demonstrated the robustness of the model via successful cross-validation using three different ADNI cohorts with varying data acquisition protocol and scanner resolutions. We also verified the generalizability of LSN on a replication AIBL dataset. Lastly, we provide an example use case that could further help clinicians identify subjects that would benefit the most from LSN model predictions. We believe this work will further motivate the exploration of multi-modal, longitudinal models that would improve the prognostic predictions and patient care in AD.

## Supporting information

S1 File Subject lists. ADNI and AIBL subjects used in the analysis.
(DOCX)

S2 File Hyperparameter search. Ranges of hyperparameter values used during the grid-search.
(DOCX)

S3 File Clinical score distributions. Clinical score distributions of different trajectories at two timepoints.
(DOCX)

S4 File Prediction performance tables. AUC and accuracy values for both tasks with all input combinations.
(DOCX)

S5 File Effect of available timepoints (duration) on prediction performance. (DOCX)

S6 File Cross-validation paradigm. (DOCX)

S7 File ADNI Acknowledgement. (DOCX)
