# Supplementary material for: Modeling and prediction of clinical symptom trajectories in Alzheimer’s disease using longitudinal data
Source: PLoS Comput Biol. 2018 Sep 14;14(9):e1006376. doi: 10.1371/journal.pcbi.1006376 (PMC6157905; doi:10.1371/journal.pcbi.1006376)
Supplement: S1 File — ADNI and AIBL subjects used in the analysis. (DOCX) [file pcbi.1006376.s001.docx]

**S1. Subject Lists**

**ADNI (Trajectory Modeling)**

023_S_0042 , 136_S_0107 , 123_S_0108 , 127_S_0112 , 027_S_0116 , 023_S_0126 , 018_S_0142 , 098_S_0160 , 010_S_0161 , 021_S_0178 , 032_S_0214 , 027_S_0256 , 021_S_0276 , 130_S_0285 , 130_S_0289 , 035_S_0292 , 031_S_0294 , 027_S_0307 , 023_S_0331 , 116_S_0361 , 131_S_0384 , 027_S_0408 , 037_S_0539 , 005_S_0546 , 027_S_0644 , 016_S_0702 , 126_S_0709 , 002_S_0729 , 068_S_0802 , 027_S_0835 , 033_S_0906 , 053_S_0919 , 033_S_0922 , 052_S_0952 , 032_S_0978 , 137_S_0994 , 127_S_1032 , 003_S_1074 , 037_S_1078 , 022_S_1097 , 003_S_1122 , 006_S_1130 , 126_S_1187 , 116_S_1243 , 129_S_1246 , 057_S_1269 , 123_S_1300 , 052_S_1346 , 137_S_1414 , 041_S_1418 , 127_S_1427 , 021_S_0626 , 014_S_0169 , 023_S_0217 , 014_S_0563 , 005_S_0572 , 014_S_0658 , 137_S_0668 , 137_S_0722 , 116_S_0752 , 137_S_0800 , 029_S_0914 , 027_S_1045 , 031_S_1066 , 002_S_1155 , 002_S_1268 , 029_S_1318 , 016_S_1326 , 052_S_1352

**ADNI (Trajectory Prediction)**

002_S_0295,002_S_0413,002_S_0619,002_S_0685,002_S_0782,002_S_0816,002_S_0938,002_S_0954,002_S_1018,002_S_1070,002_S_1261,002_S_1280,002_S_2010,002_S_2073,002_S_4171,002_S_4213,002_S_4219,002_S_4225,002_S_4229,002_S_4237,002_S_4262,002_S_4270,002_S_4447,002_S_4473,002_S_4521,002_S_4654,002_S_4746,002_S_4799,002_S_5018,002_S_5178,002_S_5230,002_S_5256,003_S_0907,003_S_0981,003_S_1057,003_S_2374,003_S_4119,003_S_4136,003_S_4288,003_S_4350,003_S_4354,003_S_4441,003_S_4555,003_S_4644,003_S_4872,003_S_4892,003_S_4900,003_S_5130,005_S_0221,005_S_0222,005_S_0223,005_S_0324,005_S_0448,005_S_0553,005_S_0602,005_S_0610,005_S_0814,005_S_1224,005_S_1341,005_S_2390,005_S_4168,005_S_4185,005_S_4707,005_S_5038,006_S_0498,006_S_0547,006_S_0675,006_S_0681,006_S_0731,006_S_4150,006_S_4153,006_S_4192,006_S_4346,006_S_4357,006_S_4363,006_S_4485,006_S_4515,006_S_4679,006_S_4713,006_S_4960,007_S_0041,007_S_0068,007_S_0070,007_S_0101,007_S_0128,007_S_0249,007_S_0293,007_S_0316,007_S_0414,007_S_0698,007_S_1206,007_S_1222,007_S_2394,007_S_4272,007_S_4387,007_S_4467,007_S_4488,007_S_4516,007_S_4568,007_S_4611,007_S_4620,007_S_4637,007_S_4911,009_S_0842,009_S_0862,009_S_1030,009_S_2208,009_S_2381,009_S_4324,009_S_4337,009_S_4359,009_S_4388,009_S_4530,009_S_4543,009_S_4612,009_S_4741,009_S_4814,009_S_4958,009_S_5000,009_S_5027,009_S_5037,009_S_5125,010_S_0067,010_S_0419,010_S_0420,010_S_0422,010_S_0472,010_S_0786,010_S_0829,010_S_0904,010_S_4345,010_S_4442,011_S_0016,011_S_0021,011_S_0022,011_S_0023,011_S_0053,011_S_0183,011_S_0241,011_S_0326,011_S_0362,011_S_0856,011_S_0861,011_S_1080,011_S_1282,011_S_2274,011_S_4075,011_S_4105,011_S_4120,011_S_4222,011_S_4235,011_S_4278,011_S_4366,011_S_4547,011_S_4827,011_S_4893,011_S_4912,012_S_0634,012_S_0637,012_S_0689,012_S_0712,012_S_0720,012_S_0803,012_S_0932,012_S_1009,012_S_1033,012_S_1133,012_S_1165,012_S_1292,012_S_1321,012_S_4012,012_S_4026,012_S_4094,012_S_4128,012_S_4188,012_S_4545,012_S_4643,012_S_4849,012_S_4987,013_S_0240,013_S_0325,013_S_0502,013_S_0575,013_S_0860,013_S_1035,013_S_1120,013_S_1186,013_S_1205,013_S_1275,013_S_4268,013_S_4395,013_S_4579,013_S_4580,013_S_4595,013_S_4616,013_S_4791,013_S_4917,013_S_4985,013_S_5137,014_S_0328,014_S_0519,014_S_0520,014_S_0548,014_S_0557,014_S_0558,014_S_1095,014_S_2185,014_S_4039,014_S_4058,014_S_4079,014_S_4080,014_S_4263,014_S_4328,014_S_4401,014_S_4576,014_S_4577,016_S_0354,016_S_0359,016_S_0538,016_S_0991,016_S_1028,016_S_1117,016_S_1121,016_S_2007,016_S_2031,016_S_4009,016_S_4121,016_S_4584,016_S_4591,016_S_4638,016_S_4646,016_S_4902,016_S_4951,016_S_4952,016_S_5007,016_S_5031,016_S_5057,018_S_0055,018_S_0080,018_S_0087,018_S_0155,018_S_0286,018_S_0335,018_S_0369,018_S_0425,018_S_0450,018_S_0633,018_S_2133,018_S_2155,018_S_2180,018_S_4257,018_S_4313,018_S_4349,018_S_4399,018_S_4400,018_S_4597,018_S_4696,018_S_4809,018_S_4868,018_S_4889,019_S_4252,019_S_4285,019_S_4293,019_S_4367,019_S_4477,019_S_4548,019_S_4549,019_S_4680,019_S_4835,019_S_5019,019_S_5242,020_S_0097,020_S_0213,020_S_0883,020_S_0899,020_S_1288,020_S_4920,021_S_0141,021_S_0159,021_S_0231,021_S_0273,021_S_0332,021_S_0337,021_S_0343,021_S_0424,021_S_0642,021_S_0647,021_S_0753,021_S_0984,021_S_1109,021_S_2077,021_S_2100,021_S_2125,021_S_2142,021_S_4245,021_S_4659,021_S_4718,022_S_0066,022_S_0096,022_S_0129,022_S_0544,022_S_0750,022_S_0961,022_S_1351,022_S_4173,022_S_4196,022_S_4266,022_S_4291,022_S_4320,022_S_4444,022_S_4805,022_S_4922,022_S_5004,023_S_0030,023_S_0031,023_S_0058,023_S_0061,023_S_0078,023_S_0081,023_S_0083,023_S_0084,023_S_0139,023_S_0376,023_S_0604,023_S_0625,023_S_0855,023_S_0926,023_S_0963,023_S_1046,023_S_1126,023_S_1247,023_S_1262,023_S_4020,023_S_4035,023_S_4115,023_S_4122,023_S_4164,023_S_4243,023_S_4448,023_S_4501,023_S_4502,023_S_4796,024_S_0985,024_S_1171,024_S_1393,024_S_2239,024_S_4084,024_S_4158,024_S_4169,024_S_4223,024_S_4280,024_S_4392,024_S_4674,024_S_4905,024_S_5054,027_S_0074,027_S_0120,027_S_0179,027_S_0403,027_S_0404,027_S_0417,027_S_0461,027_S_0485,027_S_0850,027_S_1081,027_S_1082,027_S_1213,027_S_1254,027_S_1277,027_S_1387,027_S_2183,027_S_2219,027_S_2245,027_S_2336,027_S_4729,027_S_4757,027_S_4802,027_S_4804,027_S_4869,027_S_4873,027_S_4919,027_S_4926,027_S_4936,027_S_4938,027_S_4955,027_S_4962,027_S_4964,027_S_4966,027_S_5079,027_S_5083,027_S_5093,027_S_5109,027_S_5110,027_S_5118,027_S_5127,029_S_0824,029_S_0836,029_S_0843,029_S_0845,029_S_0866,029_S_0878,029_S_0999,029_S_1056,029_S_1384,029_S_2376,029_S_2395,029_S_4279,029_S_4290,029_S_4327,029_S_4384,029_S_4385,029_S_4585,029_S_4652,031_S_0321,031_S_0351,031_S_0554,031_S_0568,031_S_0618,031_S_0830,031_S_1209,031_S_2018,031_S_2022,031_S_2233,031_S_4005,031_S_4021,031_S_4024,031_S_4029,031_S_4032,031_S_4042,031_S_4149,031_S_4194,031_S_4203,031_S_4218,031_S_4496,031_S_4721,032_S_0095,032_S_0187,032_S_0479,032_S_0677,032_S_0718,032_S_1101,032_S_1169,032_S_5289,033_S_0511,033_S_0513,033_S_0514,033_S_0516,033_S_0567,033_S_0723,033_S_0724,033_S_0725,033_S_0733,033_S_0734,033_S_0739,033_S_0741,033_S_0923,033_S_1281,033_S_1284,033_S_1285,033_S_1308,033_S_1309,033_S_4176,033_S_4177,033_S_4179,033_S_4508,033_S_5087,035_S_0033,035_S_0048,035_S_0156,035_S_0204,035_S_0341,035_S_0555,035_S_0997,035_S_2061,035_S_2074,035_S_4082,035_S_4114,035_S_4256,035_S_4414,035_S_4464,035_S_4582,035_S_4784,036_S_0576,036_S_0577,036_S_0656,036_S_0672,036_S_0673,036_S_0748,036_S_0759,036_S_0760,036_S_0813,036_S_0869,036_S_0945,036_S_0976,036_S_1001,036_S_1135,036_S_1240,036_S_2378,036_S_2380,036_S_4389,036_S_4430,036_S_4491,036_S_4538,036_S_4562,036_S_4714,036_S_4715,036_S_4736,036_S_4878,036_S_4894,036_S_4899,037_S_0150,037_S_0303,037_S_0327,037_S_0454,037_S_0467,037_S_0501,037_S_0552,037_S_0566,037_S_0588,037_S_0627,037_S_1225,037_S_1421,037_S_4001,037_S_4015,037_S_4028,037_S_4030,037_S_4071,037_S_4146,037_S_4214,041_S_0125,041_S_0282,041_S_0446,041_S_0549,041_S_1002,041_S_1010,041_S_1260,041_S_1368,041_S_1412,041_S_1425,041_S_4138,041_S_4271,041_S_4510,041_S_4874,041_S_4876,041_S_4989,041_S_5026,041_S_5078,041_S_5082,041_S_5097,041_S_5100,041_S_5131,041_S_5141,051_S_1040,051_S_1072,051_S_1123,051_S_1131,051_S_1296,051_S_1331,051_S_4929,051_S_4980,051_S_5005,052_S_0671,052_S_0951,052_S_1054,052_S_1168,052_S_1250,052_S_1251,052_S_4626,052_S_4807,052_S_4885,052_S_4944,052_S_4945,053_S_0389,053_S_0507,053_S_1044,053_S_2357,053_S_2396,053_S_4557,053_S_4578,053_S_4661,053_S_4813,053_S_5070,057_S_0464,057_S_0474,057_S_0643,057_S_0779,057_S_0818,057_S_0839,057_S_0934,057_S_0941,057_S_1007,057_S_1217,057_S_1265,057_S_1373,057_S_2398,057_S_4888,057_S_4897,062_S_0578,062_S_0690,062_S_0730,062_S_0768,062_S_1099,062_S_1182,062_S_1299,067_S_0019,067_S_0029,067_S_0038,067_S_0056,067_S_0059,067_S_0076,067_S_0077,067_S_0098,067_S_0176,067_S_0177,067_S_0284,067_S_0290,067_S_0336,067_S_0607,067_S_2195,067_S_2196,067_S_2301,067_S_2304,067_S_4054,067_S_4072,067_S_4184,067_S_4212,067_S_4310,067_S_4767,067_S_4782,067_S_4918,068_S_0109,068_S_0127,068_S_0210,068_S_0442,068_S_0473,068_S_0872,068_S_2187,068_S_2248,068_S_4061,068_S_4067,068_S_4134,068_S_4174,068_S_4217,068_S_4340,068_S_4424,070_S_5040,072_S_0315,072_S_2027,072_S_2037,072_S_2072,072_S_2083,072_S_2093,072_S_2116,072_S_2164,072_S_4007,072_S_4057,072_S_4063,072_S_4102,072_S_4103,072_S_4131,072_S_4206,072_S_4226,072_S_4383,072_S_4390,072_S_4391,072_S_4394,072_S_4445,072_S_4462,072_S_4465,072_S_4522,072_S_4539,072_S_4610,072_S_4613,072_S_4769,072_S_4871,072_S_4941,072_S_5207,073_S_0089,073_S_0311,073_S_0312,073_S_0386,073_S_0518,073_S_0746,073_S_0909,073_S_2153,073_S_2182,073_S_2190,073_S_2191,073_S_2225,073_S_2264,073_S_4155,073_S_4216,073_S_4259,073_S_4300,073_S_4311,073_S_4312,073_S_4360,073_S_4382,073_S_4393,073_S_4443,073_S_4552,073_S_4559,073_S_4614,073_S_4762,073_S_4777,073_S_4825,073_S_5023,082_S_0928,082_S_1119,082_S_1256,082_S_1377,082_S_2099,082_S_2121,082_S_2307,082_S_4090,082_S_4208,082_S_4224,082_S_4244,082_S_4339,082_S_4428,082_S_5014,082_S_5029,094_S_0434,094_S_0526,094_S_0531,094_S_0692,094_S_0711,094_S_0921,094_S_1027,094_S_1090,094_S_1164,094_S_1188,094_S_1241,094_S_1267,094_S_1293,094_S_1417,094_S_2201,094_S_2216,094_S_2238,094_S_2367,094_S_4089,094_S_4234,094_S_4434,094_S_4503,094_S_4560,094_S_4649,098_S_0171,098_S_0172,098_S_0269,098_S_0896,098_S_2047,098_S_2079,098_S_4003,098_S_4018,098_S_4215,098_S_4275,098_S_4506,099_S_0040,099_S_0051,099_S_0054,099_S_0060,099_S_0090,099_S_0111,099_S_0291,099_S_0352,099_S_0372,099_S_0470,099_S_0533,099_S_0534,099_S_0551,099_S_0880,099_S_1034,099_S_1144,099_S_2042,099_S_2063,099_S_2146,099_S_2205,099_S_4076,099_S_4086,099_S_4104,099_S_4157,099_S_4202,099_S_4205,099_S_4463,099_S_4475,099_S_4480,099_S_4498,099_S_4565,100_S_0015,100_S_0035,100_S_0047,100_S_0069,100_S_0190,100_S_0296,100_S_0995,100_S_4469,100_S_4512,100_S_4556,100_S_5096,109_S_0950,109_S_0967,109_S_1014,109_S_1114,109_S_1157,109_S_1183,109_S_1343,109_S_2200,109_S_4380,109_S_4455,109_S_4499,109_S_4531,109_S_4594,114_S_0166,114_S_0173,114_S_0374,114_S_0378,114_S_0410,114_S_0416,114_S_0458,114_S_0601,114_S_0979,114_S_1103,114_S_1106,114_S_1118,114_S_2392,114_S_4404,114_S_5047,116_S_0370,116_S_0382,116_S_0392,116_S_0487,116_S_0648,116_S_0649,116_S_0657,116_S_1232,116_S_1249,116_S_1271,116_S_1315,116_S_4010,116_S_4043,116_S_4092,116_S_4167,116_S_4175,116_S_4195,116_S_4199,116_S_4338,116_S_4453,116_S_4483,116_S_4625,116_S_4635,116_S_4855,116_S_4898,121_S_1322,123_S_0050,123_S_0072,123_S_0088,123_S_0091,123_S_0106,123_S_0113,123_S_0162,123_S_0390,123_S_2055,123_S_2363,123_S_4096,123_S_4127,123_S_4170,123_S_4526,123_S_4780,123_S_4806,126_S_0605,126_S_0606,126_S_0680,126_S_0708,126_S_0865,126_S_0891,126_S_1221,126_S_2360,126_S_2405,126_S_2407,126_S_4458,126_S_4494,126_S_4507,126_S_4514,126_S_4675,126_S_4712,126_S_4743,126_S_4891,126_S_4896,127_S_0259,127_S_0260,127_S_0393,127_S_0394,127_S_0431,127_S_0684,127_S_0754,127_S_0844,127_S_1140,127_S_1419,127_S_2213,127_S_2234,127_S_4148,127_S_4197,127_S_4198,127_S_4210,127_S_4240,127_S_4301,127_S_4500,127_S_4604,127_S_4624,127_S_4645,127_S_4844,127_S_4928,127_S_4940,127_S_5058,127_S_5067,127_S_5095,127_S_5132,128_S_0200,128_S_0230,128_S_0863,128_S_0947,128_S_1043,128_S_1088,128_S_1148,128_S_1242,128_S_1407,128_S_1408,128_S_2002,128_S_2036,128_S_2045,128_S_2123,128_S_2130,128_S_2151,128_S_2220,128_S_4553,128_S_4571,128_S_4586,128_S_4607,128_S_4636,128_S_4653,128_S_4742,128_S_4772,128_S_4832,128_S_4842,128_S_5066,129_S_0778,129_S_2332,129_S_4073,129_S_4220,129_S_4287,129_S_4369,129_S_4371,129_S_4396,129_S_4422,130_S_0102,130_S_0232,130_S_0423,130_S_0505,130_S_0783,130_S_0886,130_S_0956,130_S_0969,130_S_1200,130_S_1201,130_S_1290,130_S_1337,130_S_2391,130_S_2403,130_S_4250,130_S_4294,130_S_4343,130_S_4352,130_S_4405,130_S_4415,130_S_4417,130_S_4468,130_S_4542,130_S_4605,130_S_4641,130_S_4660,130_S_4817,130_S_4883,130_S_4925,130_S_4982,130_S_4984,130_S_4990,130_S_5006,130_S_5142,131_S_0123,131_S_0441,131_S_1389,132_S_0987,133_S_0433,133_S_0488,133_S_0525,133_S_0629,133_S_0638,133_S_0727,133_S_0771,133_S_0792,133_S_0912,133_S_0913,133_S_1170,135_S_4281,135_S_4309,135_S_4356,135_S_4406,135_S_4446,135_S_4489,135_S_4566,135_S_4598,135_S_4657,135_S_4676,135_S_4689,135_S_4722,135_S_4723,135_S_4863,135_S_5015,136_S_0086,136_S_0184,136_S_0186,136_S_0194,136_S_0195,136_S_0196,136_S_0299,136_S_0426,136_S_0429,136_S_0579,136_S_0695,136_S_0873,136_S_0874,136_S_1227,136_S_4189,136_S_4269,137_S_0158,137_S_0283,137_S_0301,137_S_0366,137_S_0443,137_S_0459,137_S_0481,137_S_0631,137_S_0669,137_S_0686,137_S_0796,137_S_0825,137_S_0972,137_S_0973,137_S_4211,137_S_4258,137_S_4299,137_S_4303,137_S_4331,137_S_4351,137_S_4466,137_S_4482,137_S_4520,137_S_4536,137_S_4587,137_S_4596,137_S_4623,137_S_4631,137_S_4632,137_S_4672,137_S_4678,137_S_4815,137_S_4816,137_S_4852,141_S_0696,141_S_0717,141_S_0767,141_S_0790,141_S_0810,141_S_0851,141_S_0852,141_S_0853,141_S_0915,141_S_0982,141_S_1004,141_S_1052,141_S_1094,141_S_1137,141_S_1152,141_S_1245,141_S_1255,141_S_1378,141_S_4053,141_S_4160,141_S_4232,141_S_4426,153_S_2109,153_S_2148,153_S_4077,153_S_4125,153_S_4133,153_S_4151,153_S_4159,153_S_4172,153_S_4372,153_S_4621,153_S_4838,941_S_1194,941_S_1197,941_S_1202,941_S_1311,941_S_4036,941_S_4100,941_S_4187,941_S_4255,941_S_4292,941_S_4365,941_S_4376,941_S_4420,941_S_4764,941_S_5124

**AIBL**

14, 17, 18, 20, 21, 22, 23, 26, 27, 28, 29, 31, 33, 38, 39, 43, 44, 50, 52, 55, 56, 57, 61, 62, 68, 75, 80, 88, 90, 111, 117, 118, 121, 126, 152, 153, 156, 186, 194, 212, 229, 232, 236, 244, 273, 277, 284, 287, 294, 310, 314, 315, 317, 331, 335, 354, 362, 365, 367, 380, 388, 390, 406, 471, 480, 482, 483, 509, 516, 518, 527, 528, 550, 551, 557, 570, 571, 572, 573, 588, 605, 609, 666, 681, 696, 697, 698, 699, 707, 716, 721, 722, 736, 737, 740, 757, 771, 784, 796, 798, 808, 814, 827, 851, 891, 904, 914, 931, 938, 942, 945, 1050, 1067, 1146, 1147, 1153, 1157
