# Supplementary material for: Modeling and prediction of clinical symptom trajectories in Alzheimer’s disease using longitudinal data
Source: PLoS Comput Biol. 2018 Sep 14;14(9):e1006376. doi: 10.1371/journal.pcbi.1006376 (PMC6157905; doi:10.1371/journal.pcbi.1006376)
Supplement: S2 File — Ranges of hyperparameter values used during the grid-search. (DOCX) [file pcbi.1006376.s002.docx]

**S2. Hyperparameter search**

The internal validation procedure searches through a reasonable set of permutation of hyperparameters to decide optimal model architecture that balances the accuracy and generalizability metrics. Below are the hyperparameter search spaces for each model used in the analysis.

Table A: Hyperparameter Grids

| **Model** | **Hyperparameters** |
| --- | --- |
| **LR** | 'C':[1e-3,5e-2,1e-2,5e-1,1e-1,1,1e1,1e2] |
| **SVM** | 'kernel':['linear',’rbf’], 'C':[1e-4,1e-3,1e-2,1e-1,1,1e1] |
| **RF** | 'n_estimators':[10,50,75,100,150], 'min_samples_split':[2,4,8] |
| **ANN** | n_layers: [2,3,4], n_hidden_nodes (per layer) = [5,10,25,50,100]  dropout = [0,0.1, 0.2], learning_rate = 1e-2, 1e-3,1e-4 |
| **LSN** | Siamese network:   - Number of hidden layers: 4 each branch (fixed for all folds)   - Number of nodes per layer: [25, 50] - Output: distance embedding   - Number of nodes: [10, 20]   Multiplicative module   - Number of hidden layers: 1 (fixed for all folds)   - Number of hidden nodes: 1 (fixed for all folds)   Concatenation and Prediction   - Number of hidden layers: 1 (fixed for all folds)   - Number of nodes: [10, 20] |
