# Supplementary material for: Modeling and prediction of clinical symptom trajectories in Alzheimer’s disease using longitudinal data
Source: PLoS Comput Biol. 2018 Sep 14;14(9):e1006376. doi: 10.1371/journal.pcbi.1006376 (PMC6157905; doi:10.1371/journal.pcbi.1006376)
Supplement: S3 File — Clinical score distributions of different trajectories at two timepoints. (DOCX) [file pcbi.1006376.s003.docx]

**S3. Clinical score distributions**

The clinical score distributions of subjects used in the analysis at two timepoints separated by trajectory membership. The substantial overlap between the distributions makes it difficult to differentiate between trajectories solely based on scores. The comparison between MMSE and ADAS-13 scales shows that the bigger score range of ADAS-13 scale allows modeling of symptom progression with higher specificity providing slow and fast decline trajectories.

| **MMSE** | **ADAS-13** |
| --- | --- |
| **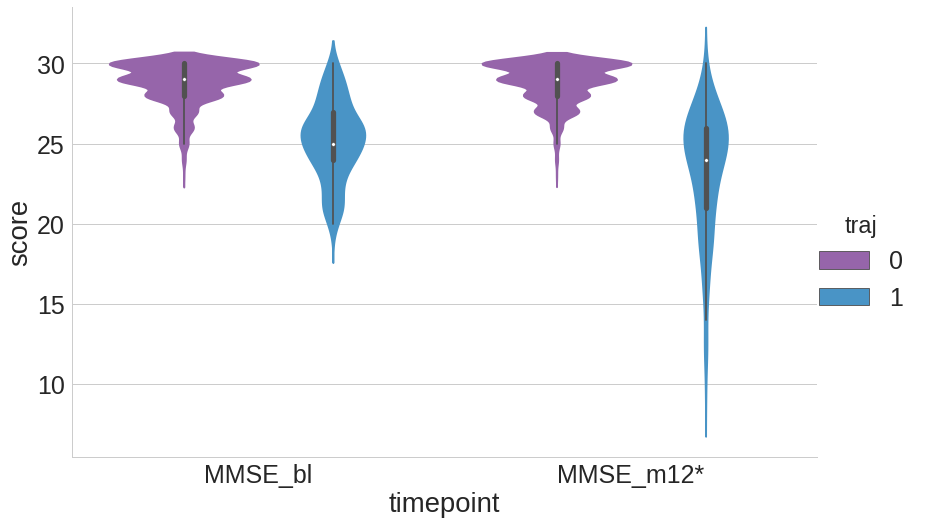** | **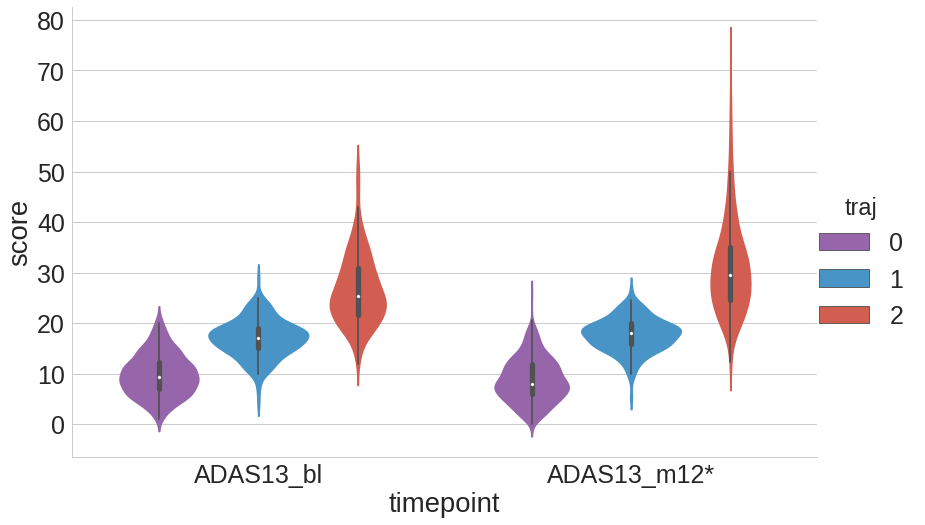** |

**Figure A:** Clinical score distributions of different trajectories at two timepoints.

*For subjects who are missing 12 month timepoint, 6 month scores are used instead.
