# Supplementary material for: Modeling and prediction of clinical symptom trajectories in Alzheimer’s disease using longitudinal data
Source: PLoS Comput Biol. 2018 Sep 14;14(9):e1006376. doi: 10.1371/journal.pcbi.1006376 (PMC6157905; doi:10.1371/journal.pcbi.1006376)
Supplement: S4 File — AUC and accuracy values for both tasks with all input combinations. (DOCX) [file pcbi.1006376.s004.docx]

**S4. Prediction performance results.**

Below are the results for two scales (MMSE, ADAS-13), two timepoints (baseline, follow-up), and three features sets (CA, CT, CA+CT). Moreover, the results are provided for all subjects and for cognitively consistent (CC) group based on the example clinical workflow. Note that only BL+follow-up, CA+CT input is applicable for LSN. Performance metrics include: accuracy (Acc), area under the ROC curve (Auc), and confusion matrix (CM).

**Table A: Predictive performance: MMSE, CA input**

**All subject**

| **MMSE** | **CA: Baseline** | | | | **CA: Baseline + Timepoint_1** | | | |
| --- | --- | --- | --- | --- | --- | --- | --- | --- |
| **Model** | **Acc** | **Auc** | **CM** | | **Acc** | **Auc** | **CM** | |
| **LR** | 0.84 (0.01) | 0.90  (0.01) | 0.88 | 0.12 | 0.90  (0.01) | 0.96  (0.01) | 0.926 | 0.074 |
|  |  |  | 0.21 | 0.79 |  |  | 0.138 | 0.862 |
| **SVM** | 0.84  (0.01) | 0.91  (0.01) | 0.87 | 0.13 | 0.90  (0.01) | 0.96  (0.01) | 0.931 | 0.069 |
|  |  |  | 0.19 | 0.81 |  |  | 0.157 | 0.843 |
| **RF** | 0.83  (0.01) | 0.90  (0.01) | 0.87 | 0.13 | 0.89  (0.01) | 0.95  (0.01) | 0.913 | 0.087 |
|  |  |  | 0.23 | 0.77 |  |  | 0.148 | 0.852 |
| **ANN** | 0.84  (0.01) | 0.91  (0.01) | 0.88 | 0.12 | 0.91  (0.01) | 0.97  (0.01) | 0.907 | 0.093 |
|  |  |  | 0.20 | 0.80 |  |  | 0.093 | 0.907 |

**Cognitively Consistent (CC) Group**

| **MMSE** | **CA: Baseline** | | | | **CA: Baseline + Timepoint_1** | | | |
| --- | --- | --- | --- | --- | --- | --- | --- | --- |
| **Model** | **Acc** | **Auc** | **CM** | | **Acc** | **Auc** | **CM** | |
| **LR** | 0.79  (0.02) | 0.85  (0.02) | 0.834 | 0.166 | 0.84  (0.02) | 0.89  (0.02) | 0.869 | 0.131 |
|  |  |  | 0.228 | 0.772 |  |  | 0.158 | 0.842 |
| **SVM** | 0.80  (0.02) | 0.88  (0.01) | 0.807 | 0.193 | 0.82  (0.02) | 0.89  (0.02) | 0.872 | 0.128 |
|  |  |  | 0.197 | 0.803 |  |  | 0.187 | 0.813 |
| **RF** | 0.81  (0.03) | 0.90  (0.02) | 0.808 | 0.192 | 0.81  (0.03) | 0.89  (0.02) | 0.829 | 0.171 |
|  |  |  | 0.204 | 0.796 |  |  | 0.161 | 0.839 |
| **ANN** | 0.80  (0.02) | 0.87  (0.02) | 0.827 | 0.173 | 0.831  (0.03) | 0.90  (0.02) | 0.803 | 0.197 |
|  |  |  | 0.207 | 0.793 |  |  | 0.114 | 0.886 |

**Table B: Predictive performance: MMSE, CT input**

**All subjects**

| **MMSE** | **CT: Baseline** | | | | **CT: Baseline + Timepoint_1** | | | |
| --- | --- | --- | --- | --- | --- | --- | --- | --- |
| **Model** | **Acc** | **Auc** | **CM** | | **Acc** | **Auc** | **CM** | |
| **LR** | 0.76  (0.01) | 0.82  (0.02) | 0.824 | 0.176 | 0.77  (0.02) | 0.83  (0.02) | 0.83 | 0.17 |
|  |  |  | 0.321 | 0.679 |  |  | 0.31 | 0.69 |
| **SVM** | 0.77  (0.01) | 0.83  (0.01) | 0.83 | 0.17 | 0.76  (0.01) | 0.82  (0.01) | 0.829 | 0.171 |
|  |  |  | 0.313 | 0.687 |  |  | 0.317 | 0.683 |
| **RF** | 0.75  (0.01) | 0.81  (0.01) | 0.761 | 0.239 | 0.76  (0.01) | 0.82  (0.01) | 0.781 | 0.219 |
|  |  |  | 0.276 | 0.724 |  |  | 0.28 | 0.72 |
| **ANN** | 0.75  (0.02) | 0.81  (0.01) | 0.829 | 0.171 | 0.75  (0.01) | 0.83  (0.02) | 0.833 | 0.167 |
|  |  |  | 0.333 | 0.667 |  |  | 0.334 | 0.666 |

**Cognitively Consistent (CC) Group**

| **MMSE** | **CT: Baseline** | | | | **CT: Baseline + Timepoint_1** | | | |
| --- | --- | --- | --- | --- | --- | --- | --- | --- |
| **Model** | **Acc** | **Auc** | **CM** | | **Acc** | **Auc** | **CM** | |
| **LR** | 0.73  (0.03) | 0.77  (0.04) | 0.671 | 0.329 | 0.70  (0.03) | 0.74  (0.03) | 0.652 | 0.348 |
|  |  |  | 0.188 | 0.812 |  |  | 0.216 | 0.784 |
| **SVM** | 0.75  (0.02) | 0.77  (0.04) | 0.698 | 0.302 | 0.70  (0.02) | 0.73  (0.03) | 0.639 | 0.361 |
|  |  |  | 0.179 | 0.821 |  |  | 0.211 | 0.789 |
| **RF** | 0.65  (0.03) | 0.74  (0.04) | 0.581 | 0.419 | 0.64  (0.03) | 0.74  (0.03) | 0.574 | 0.426 |
|  |  |  | 0.199 | 0.801 |  |  | 0.229 | 0.771 |
| **ANN** | 0.73  (0.03) | 0.75  (0.04) | 0.687 | 0.313 | 0.70  (0.04) | 0.73  (0.03) | 0.671 | 0.329 |
|  |  |  | 0.216 | 0.784 |  |  | 0.241 | 0.759 |

**Table C: Predictive performance: MMSE, CA+CT input**

**All subjects**

| **MMSE** | **CA+CT: Baseline** | | | | **CA+CT: Baseline + Timepoint_1** | | | |
| --- | --- | --- | --- | --- | --- | --- | --- | --- |
| **Model** | **Acc** | **Auc** | **CM** | | **Acc** | **Auc** | **CM** | |
| **LR** | 0.86  (0.01) | 0.93  (0.01) | 0.89 | 0.11 | 0.91  (0.01) | 0.96  (0.01) | 0.93 | 0.07 |
|  |  |  | 0.19 | 0.81 |  |  | 0.11 | 0.89 |
| **SVM** | 0.85  (0.01) | 0.93  (0.01) | 0.88 | 0.12 | 0.89  (0.01) | 0.96  (0.01) | 0.92 | 0.08 |
|  |  |  | 0.19 | 0.81 |  |  | 0.14 | 0.86 |
| **RF** | 0.85  (0.01) | 0.91  (0.01) | 0.86 | 0.14 | 0.88  (0.01) | 0.96  (0.00) | 0.88 | 0.12 |
|  |  |  | 0.15 | 0.85 |  |  | 0.11 | 0.89 |
| **ANN** | 0.84  (0.01) | 0.91  (0.01) | 0.89 | 0.11 | 0.89  (0.01) | 0.96  (0.01) | 0.92 | 0.08 |
|  |  |  | 0.23 | 0.77 |  |  | 0.14 | 0.86 |
| **LSN** | na | na | na | na | **0.94**  **(0.01)** | **0.99**  **(0.00)** | **0.94** | **0.06** |
|  |  |  | na | na |  |  | **0.06** | **0.94** |

**Cognitively Consistent (CC) Group**

| **MMSE** | **CA+CT: Baseline** | | | | **CA+CT: Baseline + Timepoint_1** | | | |
| --- | --- | --- | --- | --- | --- | --- | --- | --- |
| **Model** | **Acc** | **Auc** | **CM** | | **Acc** | **Auc** | **CM** | |
| **LR** | 0.80  (0.03) | 0.86  (0.03) | 0.819 | 0.181 | 0.85  (0.03) | 0.90  (0.02) | 0.842 | 0.158 |
|  |  |  | 0.201 | 0.799 |  |  | 0.123 | 0.877 |
| **SVM** | 0.80  (0.03) | 0.86  (0.02) | 0.801 | 0.199 | 0.82  (0.03) | 0.90  (0.02) | 0.81 | 0.19 |
|  |  |  | 0.169 | 0.831 |  |  | 0.16 | 0.84 |
| **RF** | 0.79  (0.04) | 0.85  (0.03) | 0.762 | 0.238 | 0.79  (0.02) | 0.90  (0.02) | 0.762 | 0.238 |
|  |  |  | 0.158 | 0.842 |  |  | 0.148 | 0.852 |
| **ANN** | 0.76  (0.04) | 0.811  (0.04) | 0.768 | 0.232 | 0.82  (0.04) | 0.88  (0.02) | 0.792 | 0.208 |
|  |  |  | 0.227 | 0.773 |  |  | 0.153 | 0.847 |
| **LSN** | na | na | na | na | **0.90**  **(0.01)** | **0.97**  **(0.01)** | **0.943** | **0.057** |
|  |  |  | na | na |  |  | **0.137** | **0.863** |

**Table D: Predictive performance: ADAS13, CA input**

**All subjects**

| **ADAS13** | **CA: Baseline** | | | | **CA: Baseline + Timepoint_1** | | | |
| --- | --- | --- | --- | --- | --- | --- | --- | --- |
| **Model** | **Acc** | **CM** | | | **Acc** | **CM** | | |
| **LR** | 0.81 (0.01) | 0.855 | 0.128 | 0.017 | 0.88 (0.01) | 0.921 | 0.076 | 0.004 |
|  |  | 0.241 | 0.496 | 0.263 |  | 0.21 | 0.698 | 0.09 |
|  |  | 0.053 | 0.161 | 0.788 |  | 0.003 | 0.123 | 0.875 |
| **SVM** | 0.78 (0.01) | 0.953 | 0.045 | 0.002 | 0.88 (0.01) | 0.967 | 0.031 | 0.002 |
|  |  | 0.38 | 0.406 | 0.212 |  | 0.255 | 0.597 | 0.147 |
|  |  | 0.003 | 0.081 | 0.916 |  | 0.003 | 0.028 | 0.969 |
| **RF** | 0.79 (0.01) | 0.883 | 0.104 | 0.013 | 0.87 (0.02) | 0.938 | 0.059 | 0.003 |
|  |  | 0.325 | 0.435 | 0.238 |  | 0.225 | 0.608 | 0.167 |
|  |  | 0.038 | 0.1 | 0.862 |  | 0.003 | 0.66 | 0.931 |
| **ANN** | 0.78 (0.00) | 0.88 | 0.109 | 0.012 | 0.82 (0.00) | 0.893 | 0.1 | 0.007 |
|  |  | - | - | - |  | - | - | - |
|  |  | 0.101 | 0.224 | 0.671 |  | 0.03 | 0.247 | 0.724 |

**Cognitively Consistent (CC) Group**

| **ADAS13** | **CA: Baseline** | | | | **CA: Baseline + Timepoint_1** | | | |
| --- | --- | --- | --- | --- | --- | --- | --- | --- |
| **Model** | **Acc** | **CM** | | | **Acc** | **CM** | | |
| **LR** | 0.58 (0.05) | 0.538 | 0.422 | 0.04 | 0.69 (0.04) | 0.631 | 0.343 | 0.024 |
|  |  | 0.166 | 0.699 | 0.133 |  | 0.18 | 0.748 | 0.07 |
|  |  | 0.046 | 0.385 | 0.568 |  | 0 | 0.317 | 0.683 |
| **SVM** | 0.64 (0.04) | - | - | - | 0.70 (0.03) | - | - | - |
|  |  | 0.296 | 0.562 | 0.141 |  | 0.212 | 0.621 | 0.166 |
|  |  | 0 | 0.136 | 0.864 |  | 0 | 0.09 | 0.91 |
| **RF** | 0.63 (0.04) | 0.541 | 0.402 | 0.056 | 0.69 (0.03) | 0.751 | 0.234 | 0.014 |
|  |  | 0.259 | 0.595 | 0.145 |  | 0.224 | 0.624 | 0.154 |
|  |  | 0.031 | 0.209 | 0.76 |  | 0 | 0.154 | 0.846 |
| **ANN** | 0.46 (0.03) | 0.623 | 0.352 | 0.025 | 0.50 (0.03) | 0.555 | 0.419 | 0.024 |
|  |  | - | - | - |  | - | - | - |
|  |  | 0.112 | 0.485 | 0.4 |  | 0.054 | 0.487 | 0.458 |

**Table E: Predictive performance: ADAS13, CT input**

**All subjects**

| **ADAS13** | **CT: Baseline** | | | | **CT: Baseline + Timepoint_1** | | | |
| --- | --- | --- | --- | --- | --- | --- | --- | --- |
| **Model** | **Acc** | **CM** | | | **Acc** | **CM** | | |
| **LR** | 0.68 (0.01) | 0.725 | 0.157 | 0.118 | 0.67 (0.01) | 0.729 | 0.158 | 0.112 |
|  |  | - | - | - |  | - | - | - |
|  |  | 0.184 | 0.186 | 0.634 |  | 0.2 | 0.177 | 0.622 |
| **SVM** | 0.61 (0.02) | 0.764 | 0.146 | 0.089 | 0.60 (0.01) | 0.772 | 0.149 | 0.082 |
|  |  | 0.518 | 0.183 | 0.302 |  | 0.502 | 0.18 | 0.319 |
|  |  | 0.157 | 0.185 | 0.658 |  | 0.168 | 0.174 | 0.658 |
| **RF** | 0.67  (0.01) | 0.683 | 0.163 | 0.153 | 0.66 (0.01) | 0.695 | 0.163 | 0.142 |
|  |  | - | - | - |  | - | - | - |
|  |  | 0.191 | 0.175 | 0.637 |  | 0.205 | 0.171 | 0.626 |
| **ANN** | 0.66 (0.01) | 0.767 | 0.14 | 0.092 | 0.65 (0.02) | 0.774 | 0.147 | 0.078 |
|  |  | 0.565 | 0.19 | 0.245 |  | 0.527 | 0.134 | 0.338 |
|  |  | 0.223 | 0.189 | 0.589 |  | 0.193 | 0.19 | 0.616 |

**Cognitively Consistent (CC) Group**

| **ADAS13** | **CT: Baseline** | | | | **CT: Baseline + Timepoint_1** | | | |
| --- | --- | --- | --- | --- | --- | --- | --- | --- |
| **Model** | **Acc** | **CM** | | | **Acc** | **CM** | | |
| **LR** | 0.37 (0.02) | 0.371 | 0.498 | 0.131 | 0.40 (0.02) | 0.393 | 0.464 | 0.143 |
|  |  | - | - | - |  | - | - | - |
|  |  | 0.162 | 0.429 | 0.407 |  | 0.11 | 0.446 | 0.441 |
| **SVM** | 0.42 (0.03) | 0.408 | 0.492 | 0.099 | 0.44 (0.02) | 0.389 | 0.466 | 0.143 |
|  |  | 0.172 | 0.426 | 0.403 |  | 0.256 | 0.486 | 0.259 |
|  |  | 0.155 | 0.441 | 0.4 |  | 0.099 | 0.429 | 0.471 |
| **RF** | 0.36 (0.02) | 0.315 | 0.478 | 0.208 | 0.37 (0.03) | 0.35 | 0.451 | 0.197 |
|  |  | - | - | - |  | - | - | - |
|  |  | 0.175 | 0.417 | 0.407 |  | 0.119 | 0.461 | 0.418 |
| **ANN** | 0.36 (0.03) | 0.381 | 0.474 | 0.146 | 0.38 (0.03) | 0.381 | 0.49 | 0.127 |
|  |  | - | - | - |  | - | - | - |
|  |  | 0.196 | 0.419 | 0.382 |  | 0.152 | 0.435 | 0.413 |

**Table F: Predictive performance: ADAS13, CA+CT input**

**All subjects**

| **ADAS13** | **CA+CT: Baseline** | | | | **CA+CT: Baseline + Timepoint_1** | | | |
| --- | --- | --- | --- | --- | --- | --- | --- | --- |
| **Model** | **Acc** | **CM** | | | **Acc** | **CM** | | |
| **LR** | 0.81  (0.01) | 0.85 | 0.13 | 0.02 | 0.84  (0.01) | 0.89 | 0.1 | 0.01 |
|  |  | 0.28 | 0.55 | 0.17 |  | 0.30 | 0.61 | 0.09 |
|  |  | 0.04 | 0.17 | 0.79 |  | 0.02 | 0.15 | 0.83 |
| **SVM** | 0.78  (0.01) | 0.92 | 0.07 | 0.01 | 0.84  (0.01) | 0.93 | 0.06 | 0.01 |
|  |  | 0.38 | 0.40 | 0.22 |  | 0.27 | 0.53 | 0.19 |
|  |  | 0.01 | 0.09 | 0.90 |  | 0.01 | 0.07 | 0.92 |
| **RF** | 0.78  (0.01) | 0.78 | 0.17 | 0.05 | 0.84  (0.01) | 0.82 | 0.15 | 0.03 |
|  |  | 0.3 | 0.46 | 0.24 |  | 0.13 | 0.75 | 0.12 |
|  |  | 0.04 | 0.14 | 0.82 |  | 0.01 | 0.10 | 0.89 |
| **ANN** | 0.79  (0.01) | 0.795 | 0.466 | 0.041 | 0.80  (0.01) | 0.887 | 0.108 | 0.008 |
|  |  | - | - | - |  | 0.336 | 0.397 | 0.265 |
|  |  | 0.036 | 0.148 | 0.816 |  | 0.029 | 0.177 | 0.794 |
| **LSN** | na | na | na | na | **0.91**  **(0.01)** | **0.96** | **0.04** | **0.00** |
|  |  | na | na | na |  | **0.18** | **0.71** | **0.11** |
|  |  | na | na | na |  | **0.00** | **0.05** | **0.95** |

**Cognitively Consistent (CC) Group**

| **ADAS13** | **CA+CT: Baseline** | | | | **CA+CT: Baseline + Timepoint_1** | | | |
| --- | --- | --- | --- | --- | --- | --- | --- | --- |
| **Model** | **Acc** | **CM** | | | **Acc** | **CM** | | |
| **LR** | 0.56 (0.04) | 0.533 | 0.432 | 0.035 | 0.59 (0.04) | 0.532 | 0.406 | 0.062 |
|  |  | - | - | - |  | 0.235 | 0.695 | 0.07 |
|  |  | 0.024 | 0.416 | 0.56 |  | 0.029 | 0.382 | 0.589 |
| **SVM** | 0.64 (0.04) | 0.752 | 0.248 | 0 | 0.63 (0.03) | 0.608 | 0.351 | 0.042 |
|  |  | 0.239 | 0.593 | 0.167 |  | 0.181 | 0.616 | 0.205 |
|  |  | 0.028 | 0.214 | 0.758 |  | 0.049 | 0.289 | 0.663 |
| **RF** | 0.49 (0.03) | 0.416 | 0.491 | 0.092 | 0.58 (0.02) | 0.445 | 0.461 | 0.092 |
|  |  | 0.183 | 0.617 | 0.2 |  | 0.087 | 0.797 | 0.116 |
|  |  | 0.019 | 0.391 | 0.59 |  | 0.022 | 0.28 | 0.697 |
| **ANN** | 0.47 (0.03) | 0.427 | 0.488 | 0.084 | 0.52 (0.04) | 0.505 | 0.438 | 0.056 |
|  |  | - | - | - |  | 0.208 | 0.54 | 0.251 |
|  |  | 0.038 | 0.405 | 0.557 |  | 0.055 | 0.424 | 0.519 |
| **LSN** | na | na | na | na | **0.76 (0.02)** | **0.797** | **0.188** | **0.014** |
|  |  | na | na | na |  | **0.159** | **0.713** | **0.128** |
|  |  | na | na | na |  | **0** | **0.137** | **0.863** |

**Table G: Predictive performance: AIBL, CA+CT input**

**AIBL Perf (All subjects)**

| **MMSE** | **CA+CT: Baseline** | | | | **CA+CT: Baseline + Timepoint_1** | | | |
| --- | --- | --- | --- | --- | --- | --- | --- | --- |
| **Model** | **Acc** | **Auc** | **CM** | | **Acc** | **Auc** | **CM** | |
| **LR** | 0.812  (0.01) | 0.859  (0.00) | 0.933 | 0.067 | 0.857  (0.00) | 0.835  (0.02) | 0.862 | 0.138 |
|  |  |  | 0.056 | 0.439 |  |  | - | - |
| **SVM** | 0.829  (0.00) | 0.863  (0.00) | 0.932 | 0.068 | 0.855  (0.00) | 0.503  (0.00) | 0.855 | 0.145 |
|  |  |  | 0.532 | 0.468 |  |  | - | - |
| **RF** | 0.815  (0.01) | 0.857  (0.00) | 0.947 | 0.053 | 0.871  (0.01) | 0.803  (0.02) | 0.912 | 0.088 |
|  |  |  | 0.550 | 0.450 |  |  | 0.417 | 0.583 |
| **ANN** | 0.856  (0.01) | 0.851  (0.01) | 0.918 | 0.082 | 0.846  (0.02) | 0.835  (0.01) | 0.886 | 0.114 |
|  |  |  | 0.393 | 0.607 |  |  | - | - |
| **LSN** | na | na | na | na | **0.724**  **(0.01)** | **0.883**  **(0.02)** | **0. 989** | **0. 011** |
|  |  |  | na | na |  |  | **0. 737** | **0. 263** |
