# Supplementary material for: Modeling and prediction of clinical symptom trajectories in Alzheimer’s disease using longitudinal data
Source: PLoS Comput Biol. 2018 Sep 14;14(9):e1006376. doi: 10.1371/journal.pcbi.1006376 (PMC6157905; doi:10.1371/journal.pcbi.1006376)
Supplement: S5 File — (DOCX) [file pcbi.1006376.s005.docx]

**S5. Effect of available timepoints (duration) on prediction performance**

The use of variable number of clinical timepoints per subject based on the availability during trajectory assignment (groundtruth) impacts the predictive performance. The stratification of performance based on last available timepoint for a given subject yielded 605, 510 subjects for 18 to 36 months (near future) and 48 to 72 month (distant future) spans, respectively. The predictive performance worsens for subjects with available timepoints between 48 and 72 months. This difference in the performance is largest with CA input and lowest with CA+CT input.

| **Dataset** | **MMSE** | **ADAS-13** |
| --- | --- | --- |
| **ADNI** | 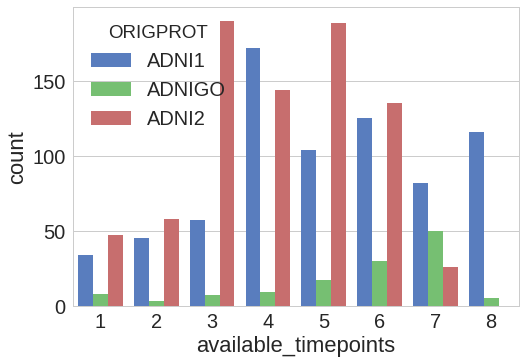 | 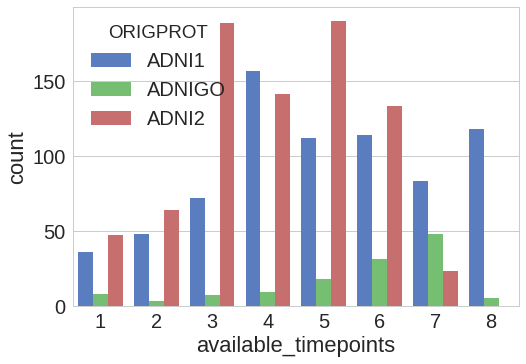 |
| **AIBL** | 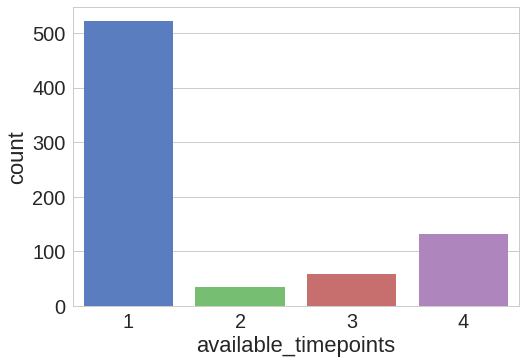 |  |

**Figure A:** Distribution of number of available timepoints with clinical score data.


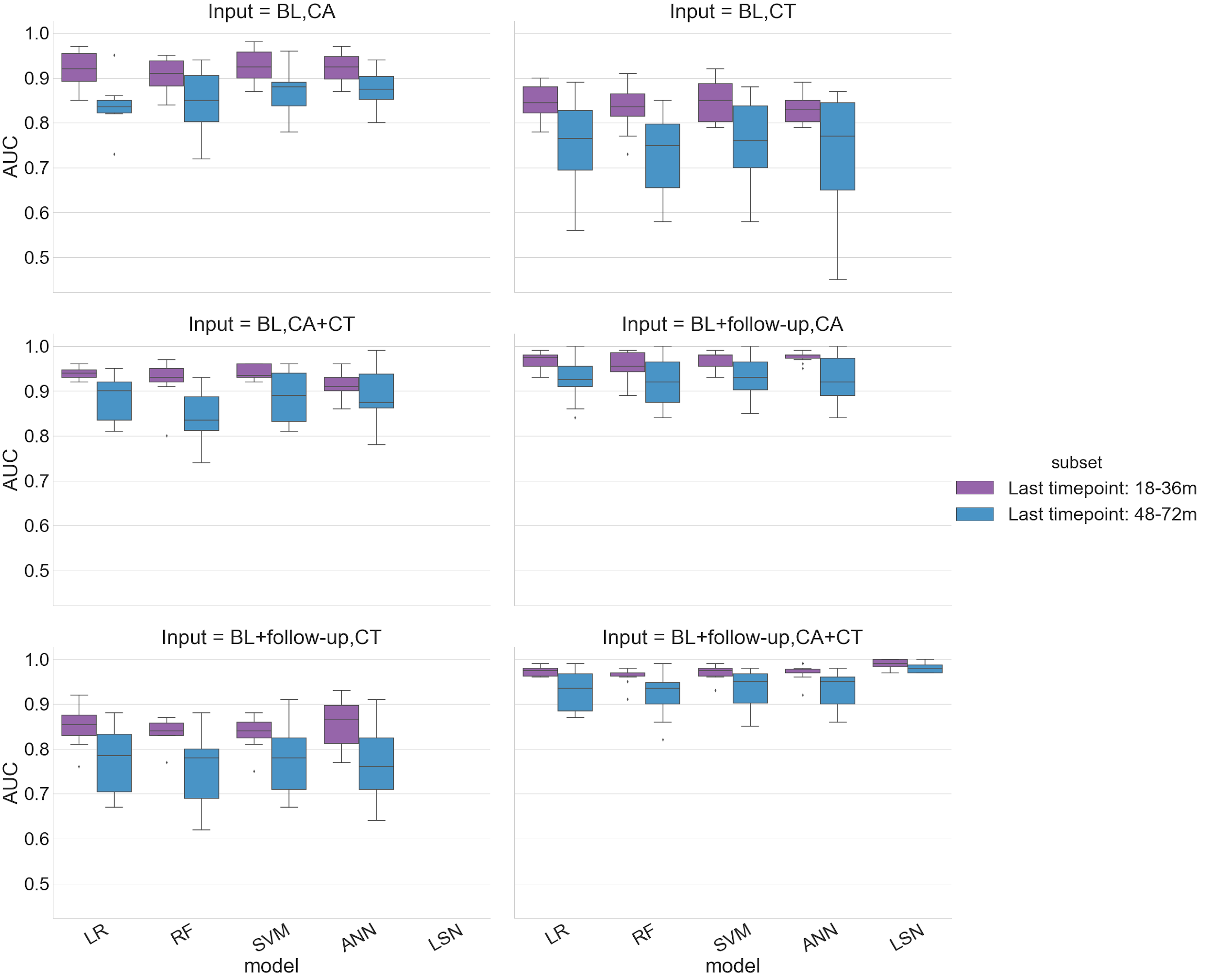


**Figure B: Effect of available timepoints on predictive performance (MMSE).** The results for 2x3 combinations of based on {baseline, follow-up} timepoints and {CA, CT, CA+CT} features. Note that only BL+follow-up, CA+CT input is applicable for LSN.

**
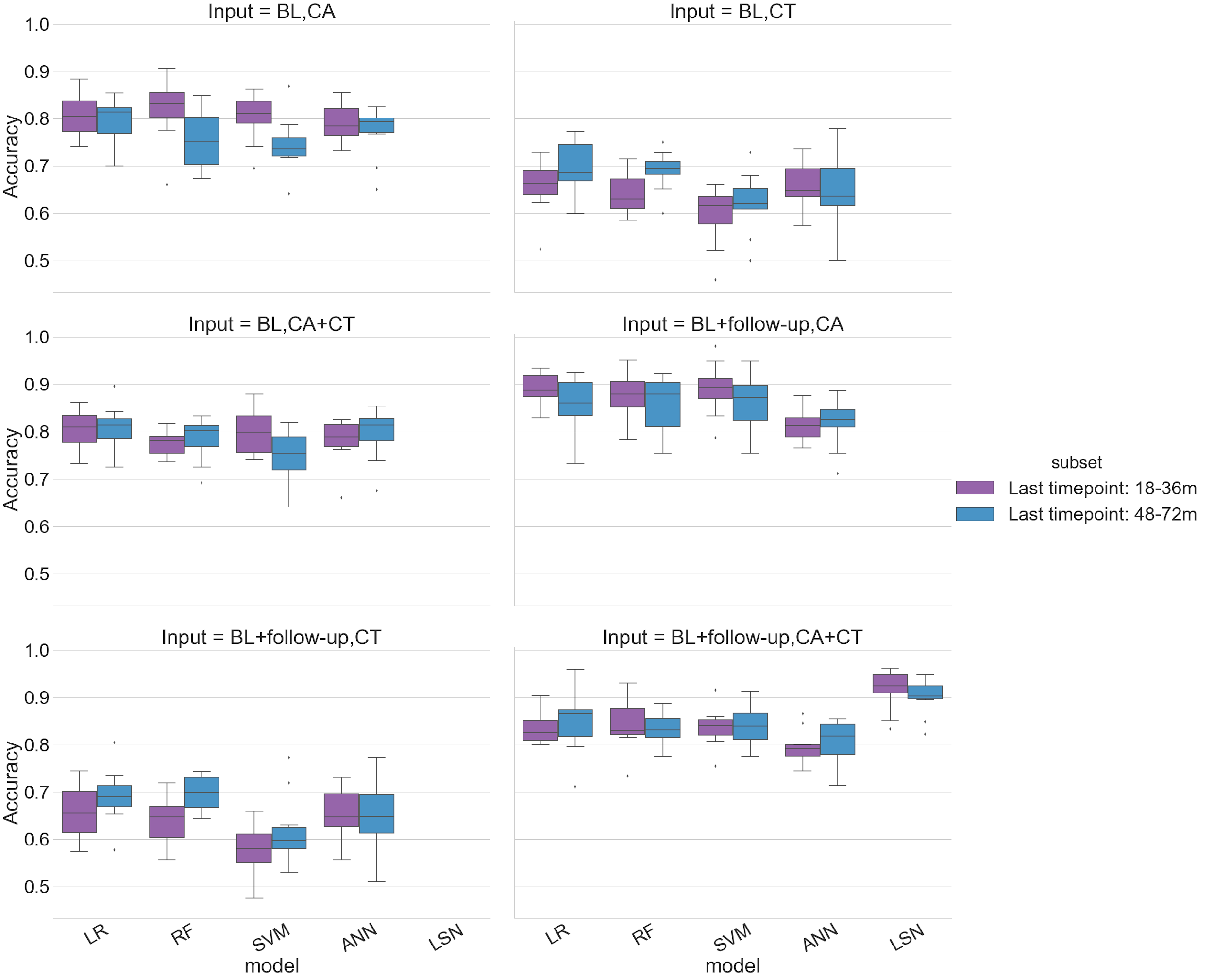
**

**Figure C: Effect of available timepoints on predictive performance (ADAS-13).** The results for 2x3 combinations of based on {baseline, follow-up} timepoints and {CA, CT, CA+CT} features. Note that only BL+follow-up, CA+CT input is applicable for LSN.
