# Supplementary material for: Modeling and prediction of clinical symptom trajectories in Alzheimer’s disease using longitudinal data
Source: PLoS Comput Biol. 2018 Sep 14;14(9):e1006376. doi: 10.1371/journal.pcbi.1006376 (PMC6157905; doi:10.1371/journal.pcbi.1006376)
Supplement: S6 File — (DOCX) [file pcbi.1006376.s006.docx]

**S6. K-fold nested cross-validation procedure**

In a K-fold nested cross-validation paradigm (see Fig. S6.1), subjects are randomly divided into K subsets. Then K-1 subsets are chosen as a training set and the remaining subset is held-out as a test set. Subsequently, The samples from each train subset are further divided into j inner folds to define and evaluate various data preprocessing operations, including feature selection, data normalization, and hyperparameter configuration. The validation subset within the inner folds is used to evaluate the performance generalizability of each model architecture. The hyperparameters (e.g. L1 penalty, number of layers, hidden nodes etc.) of any ML model are essentially “tuning knobs” that adjust the complexity of model. For ANNs, with a large number of hidden nodes, the model is able to handle more complex relationships within the input allowing more accurate predictions. However at the same time, it is also likely to overfit to the training data and offer poor performance on the test data. The internal validation procedure searches through the grid of hyperparameters to decide optimal model architecture that balances the accuracy and overfitting metrics. The nested validation set within the train set prevents double-dipping issues that may exaggerate the model performance. This optimal model (i.e. top performing hyper-parameter configuration) is then used to evaluate performance on held-out test data. This process is then repeated K times to iterate through all combination of train and test sets.

**
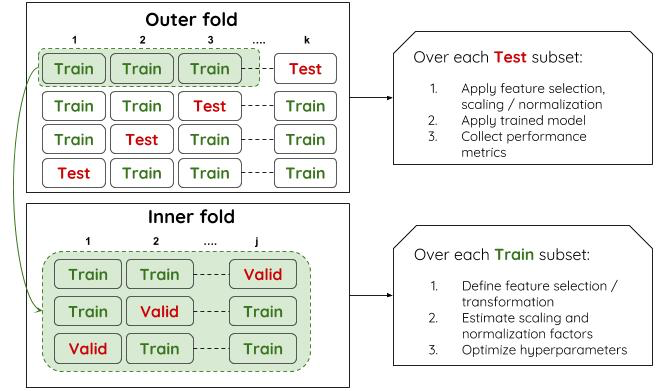
**

**Figure A: K-fold nested cross-validation.** Subjects are randomly divided into K subsets. Then K-1 subsets are chosen as a training set and the remaining subset is held-out as a test set. Subsequently, The samples from each train subset are further divided into j inner folds to define and evaluate various data preprocessing operations, including feature selection, data normalization, and hyperparameter configuration. The validation subset within the inner folds is used to evaluate the performance generalizability of each model architecture. The optimal model is then used to evaluate performance on held-out test data.
